# “Seed-Milarity” Confers to hsa-miR-210 and hsa-miR-147b Similar Functional Activity

**DOI:** 10.1371/journal.pone.0044919

**Published:** 2012-09-13

**Authors:** Thomas Bertero, Sébastien Grosso, Karine Robbe-Sermesant, Kevin Lebrigand, Imene-Sarah Hénaoui, Marie-Pierre Puisségur, Sandra Fourre, Laure-Emmanuelle Zaragosi, Nathalie M. Mazure, Gilles Ponzio, Bruno Cardinaud, Pascal Barbry, Roger Rezzonico, Bernard Mari

**Affiliations:** 1 Institut de Pharmacologie Moléculaire et Cellulaire (IPMC), Centre National de la Recherche Scientifique, CNRS UMR 7275, Sophia Antipolis, France; 2 University of Nice Sophia-Antipolis, Nice, France; 3 Centre de Physiopathologie de Toulouse-Purpan, Institut National de la Santé et de la recherche Médicale INSERM U563, Toulouse, France; 4 Institute for Research on Cancer and Ageing (IRCAN), Institut National de la Santé et de la recherche Médicale INSERM U1081, Centre National de la Recherche Scientifique CNRS UMR 7284, Nice, France; 5 Institut National de la Santé et de la recherche Médicale, INSERM U1035, Université Victor Segalen, Bordeaux, France; French National Center for Scientific Research - Institut de biologie moléculaire et cellulaire, France

## Abstract

Specificity of interaction between a microRNA (miRNA) and its targets crucially depends on the seed region located in its 5′-end. It is often implicitly considered that two miRNAs sharing the same biological activity should display similarity beyond the strict six nucleotide region that forms the seed, in order to form specific complexes with the same mRNA targets. We have found that expression of hsa-miR-147b and hsa-miR-210, though triggered by different stimuli (*i.e.* lipopolysaccharides and hypoxia, respectively), induce very similar cellular effects in term of proliferation, migration and apoptosis. Hsa-miR-147b only shares a “minimal” 6-nucleotides seed sequence with hsa-miR-210, but is identical with hsa-miR-147a over 20 nucleotides, except for one base located in the seed region. Phenotypic changes induced after heterologous expression of miR-147a strikingly differ from those induced by miR-147b or miR-210. In particular, miR-147a behaves as a potent inhibitor of cell proliferation and migration. These data fit well with the gene expression profiles observed for miR-147b and miR-210, which are very similar, and the gene expression profile of miR-147a, which is distinct from the two others. Bioinformatics analysis of all human miRNA sequences indicates multiple cases of miRNAs from distinct families exhibiting the same kind of similarity that would need to be further characterized in terms of putative functional redundancy. Besides, it implies that functional impact of some miRNAs can be masked by robust expression of miRNAs belonging to distinct families.

## Introduction

MicroRNAs (miRNAs) represent an important class of short RNAs known to play a major role in regulation of gene expression and associated with many biological functions. MiRNAs are derived from primary transcripts called pri-miRNAs. The current model of maturation includes primary nuclear cleavage of pri-miRNAs by the RNase III endonuclease Drosha, which liberates pre-miRNA hairpins. Hairpins are exported from the nucleus to the cytoplasm, where they are cleaved by Dicer, another RNase III endonuclease [1], [2]. Dicer generates short RNA sequences of about 22-nucleotides. MiRNAs are then assembled with proteins of the Argonaute family (Ago 1–4) into a ribonucleoprotein complex (miRISC) [3], [4]. In the current molecular framework, mature miRNAs, charged into the miRISC, interact with complementary site(s), often located in the 3′-untranslated region (UTR) of a putative target mRNA leading to repression of protein synthesis, often associated with mRNA degradation [5], [6], [7]. The interaction between a given miRNA and its cognate targets stems from a short stretch of 6–8 nucleotides located 5′ of the miRNA, termed the “seed sequence” [8], [9], [10]. One miRNA can theoretically target hundreds of mRNAs. Because several miRNAs can also target the same transcript, the miRNA regulatory network appears amazingly complex.

Several computational algorithms have been developed to predict transcripts which are targeted by miRNAs, the most popular being TargetScan [8], miRanda [11], microCosm Targets [12], PicTar [13], RNA22 [14], EIMMO [15], TargetSpy [16] or PACCMIT [17], [18]. Although most of them use the “seed match” as the main rule, these methods often lead to very distinct predictions. Some reasons for this imperfect overlap include the precise treatment of the seed match, in particular concerning the first target nucleotide (nt) opposite to the first miRNA nucleotide, the allowance of GU wobble pairs, the length of the seed match (6, 7 or 8 nt), the potential contribution of 3′-supplementary pairing, the degree of conservation of the site across species, the 3′UTR context or the use of different 3′UTR database [6], [7]. This clearly illustrates the limits of pure *in silico* approaches and plainly justifies an experimental verification of these predictions. Incorporation of experimental data in the pipeline of analysis can be useful in order to take into account important parameters, such as the expression of the transcripts in a specific cellular context.

Recent advances using high-throughput sequencing of RNAs have confirmed the wide range of miRNA action *in vivo* [19], [20]. MiRISC complexes can relocate target mRNAs to specialized compartments, such as the P-bodies, where translation blockade or mRNA decay occur [5]. Destabilization of mRNA induced by miRNA is substantiated by many studies. Combined with computational predictions, measurement of expression profiles for mRNAs thus represents a powerful approach to identify functional miRNA-target relationships [21], [22], [23], [24], [25], [26]. Importantly, proteomics studies [24], [27], mRNA translation profiles [28] and more recent ribosome profiling through deep sequencing of ribosome-protected mRNA fragments [29] have demonstrated that changes in mRNA levels closely reflects the impact of miRNAs on gene expression, suggesting a close relationship between miRNA-mediated translation blockade and mRNA decay, at least in mammals.

We and others have developed computational tools to explore the influence of miRNAs on gene expression profiles [30], [31], [32], [33]. Our recent bioinformatics tool MiRonTop provides the possibility to screen and compare miRNA signatures using different target prediction tools and calculate enrichment scores according to the spatial distribution of predicted targets sites along the transcript, increasing the sensitivity of the research in order to define a set of miRNA targets associated with a selected experiment [33].

We used this tool to explore a large data set of miRNA-overexpressed microarrays experiments in order to identify miRNAs with overlapping regulatory functions. We found a close relationship between two miRNAs from distinct families, hsa-miR-147b and hsa-miR-210, but sharing the same “minimal” seed sequence (2–7): UGUGCG. Interestingly, hsa-miR-147b has a close homolog, hsa-miR-147a which differs only by a 1 nt substitution in the seed sequence (UGUGUG). This preliminary observation led us to carefully explore the targetome of these 3 miRNAs with the aim to better define the important pairing and molecular rules controlling their binding and to investigate their cellular function.

## Results

### Identification of miRNAs with Related Function by Data Set Exploration of Microarrays Experiments

Data set exploration of miRNA-overexpressed microarrays experiments was performed in order to identify miRNAs with overlapping regulatory functions. For that purpose, we used a set of >50 experiments in which ∼20 miRNAs were overexpressed in different cell types and analyzed using microarrays, as previously described [25], [26], [34], [35]. We took advantage of our recently-developed MiRonTop tool [33] which detects potential overrepresentation of specific miRNAs targets across an experimental gene set using different target prediction tools and focused our attention on the identification of miRNAs with similar enrichment scores but relatively divergent sequences. Interestingly, we found a co-enrichment of hsa-miR-147b and hsa-miR-210 predicted targets in several experiments performed in different hsa-miR-210 transfected cell types (data deposited in the NCBI Gene Expression Omnibus under serie GSE33247 and data not shown) using several algorithms including for instance TargetScan and microCosm.

### Molecular Characteristics and *in silico* Target Identification of miR-210 and miR-147 Family

**Figure 1:**
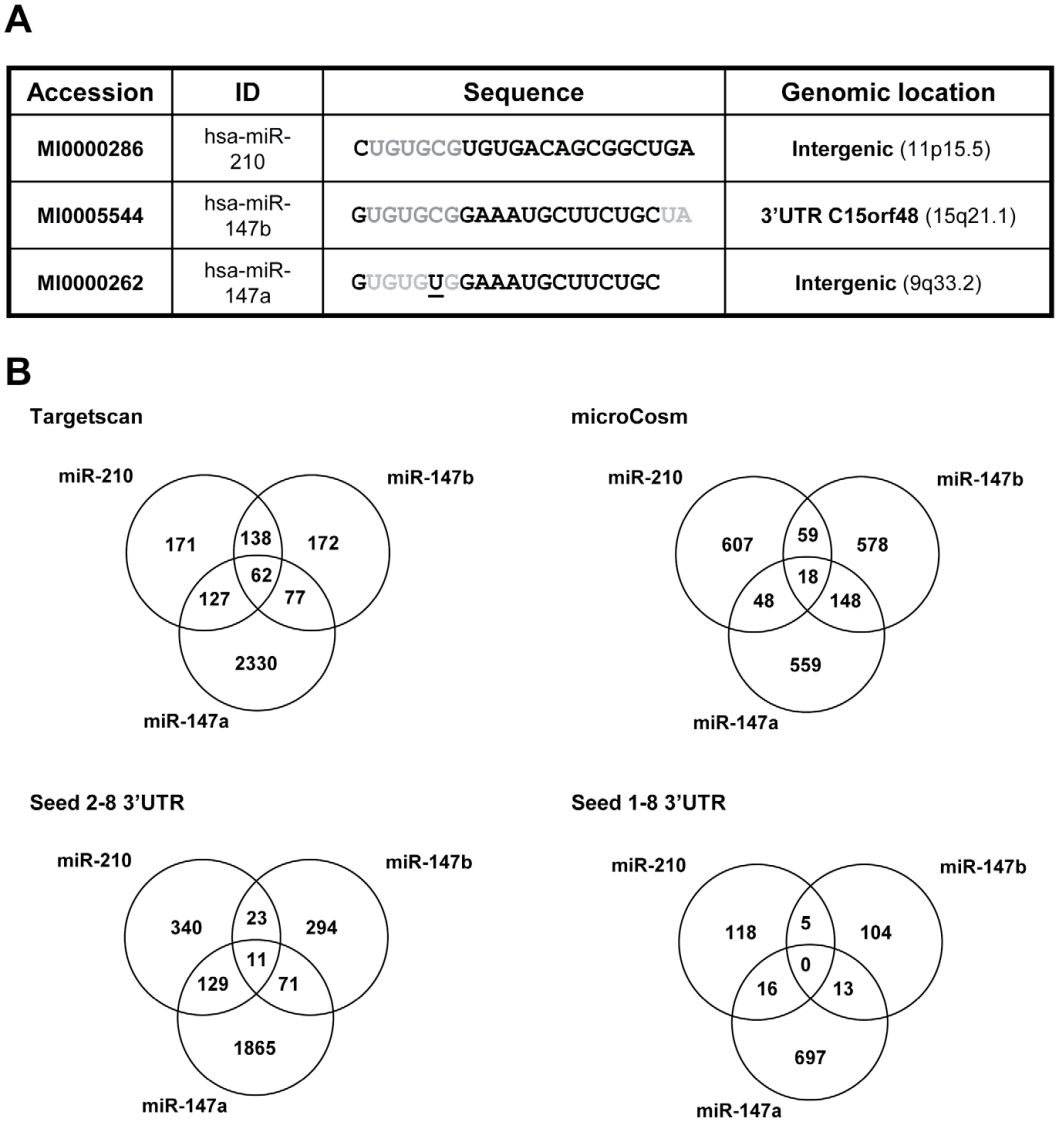
*In silico* prediction of miR-210 and miR-147 family targets. A) Alignment of miR-210, miR-147a and miR-147b mature sequences. The seed region is indicated in grey. The 1nt-substitution in miR-147a seed is underlined. B) Venn diagram summarizing the predictive common targets of miR-210 and miR-147 family members using different bioinformatics prediction tools: TargetScan, microCosm and a search of a 7 nt complementary seed 2–8 sequences in 3′UTR using our laboratory-made tool “MicroCible” [25] (http://www.microarray.fr:8080/merge/index follow the link to microRNA and Bioinformatic tools).

While hsa-miR-210 is the unique member of its family, hsa-miR-147b sequence is closely related to hsa-miR-147a and differs only with a 1 nt substitution in the seed sequence (Figure 1A). By contrast, hsa-miR-210 and hsa-miR-147b possessed an identical seed corresponding to the hexamer UGUGCG but totally divergent sequences otherwise. The use of several popular *in silico* miRNA targets prediction tools such as TargetScan [8] or microCosm [12] or a search for complementary 7 nt seed sequence in the 3′UTR indicated heterogeneous data concerning potential mRNA targeting overlap between these 3 miRNAs (Figure 1B and Figure S1A).

### Identification of miR-210, miR-147a and miR-147b Targets by mRNA Profiling of A549 Cells Overexpressing Pre-miRNAs

**Figure 2:**
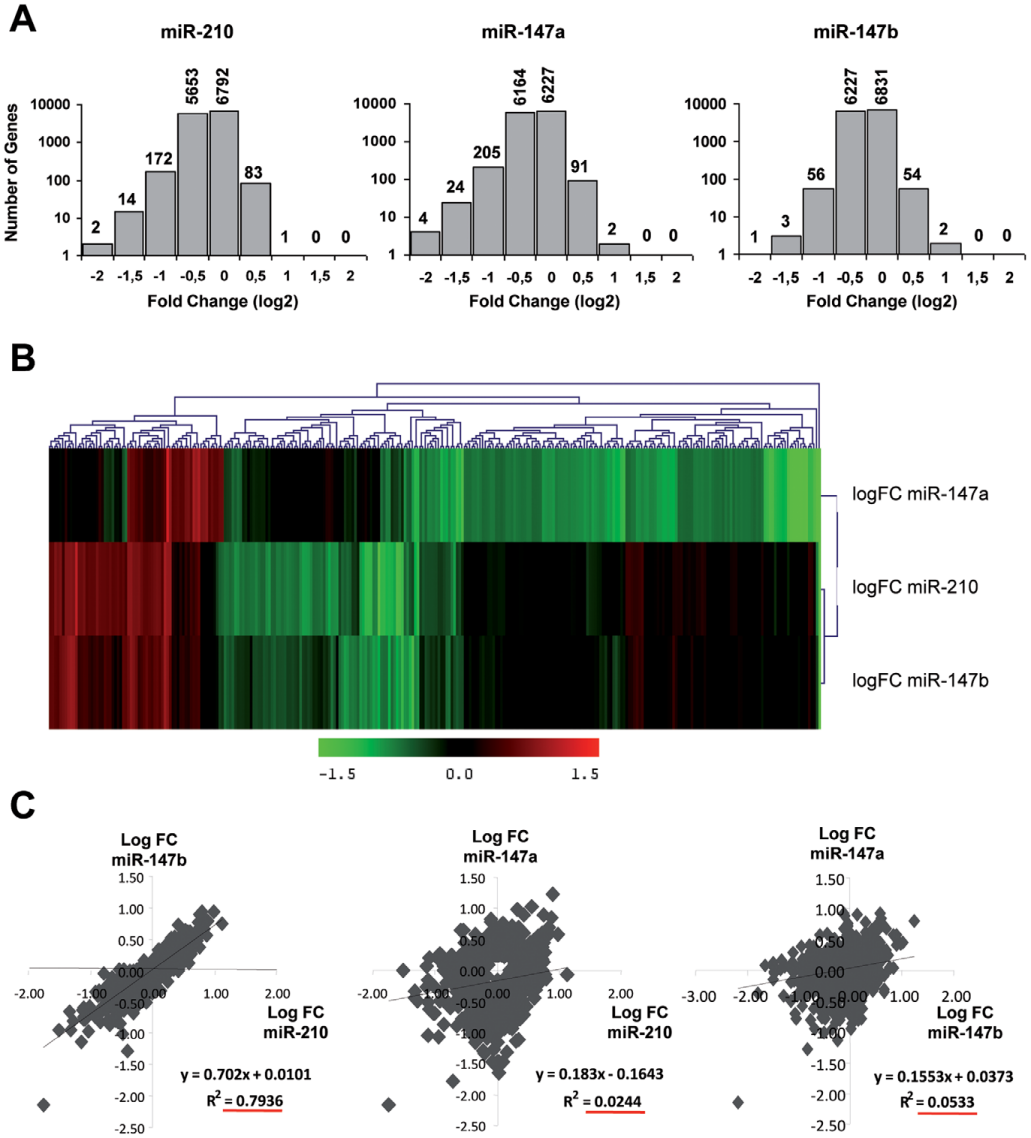
Global effect of miR-210 and miR-147 family members over-expression on transcriptome in the A549 cell line. A549 cells were transfected with synthetic pre-miR-210/147a/147b (10 nM) or with negative pre-miRNA (n = 3). RNA samples were harvested 48h post-transfection and mRNA profiles were determined with pan genomic arrays. A) Distribution of log2fold changes between each miRNA and the negative control. B) Heatmap comparing normalized log2 of the ratio between miR-210 or miR-147 family members and miR-Neg. Distance was measured using the Manhattan distance on the matrix of the log2 (ratio) and classification was performed using a complete agglomeration method. C) Correlation between the mRNA modulations mediated by the 3 different miRNAs. The correlation coefficient (R^2^) was calculated using log2 fold changes from top modulated genes (Adj.pValue <0.05 corresponding to 1098 genes).

**Figure 3:**
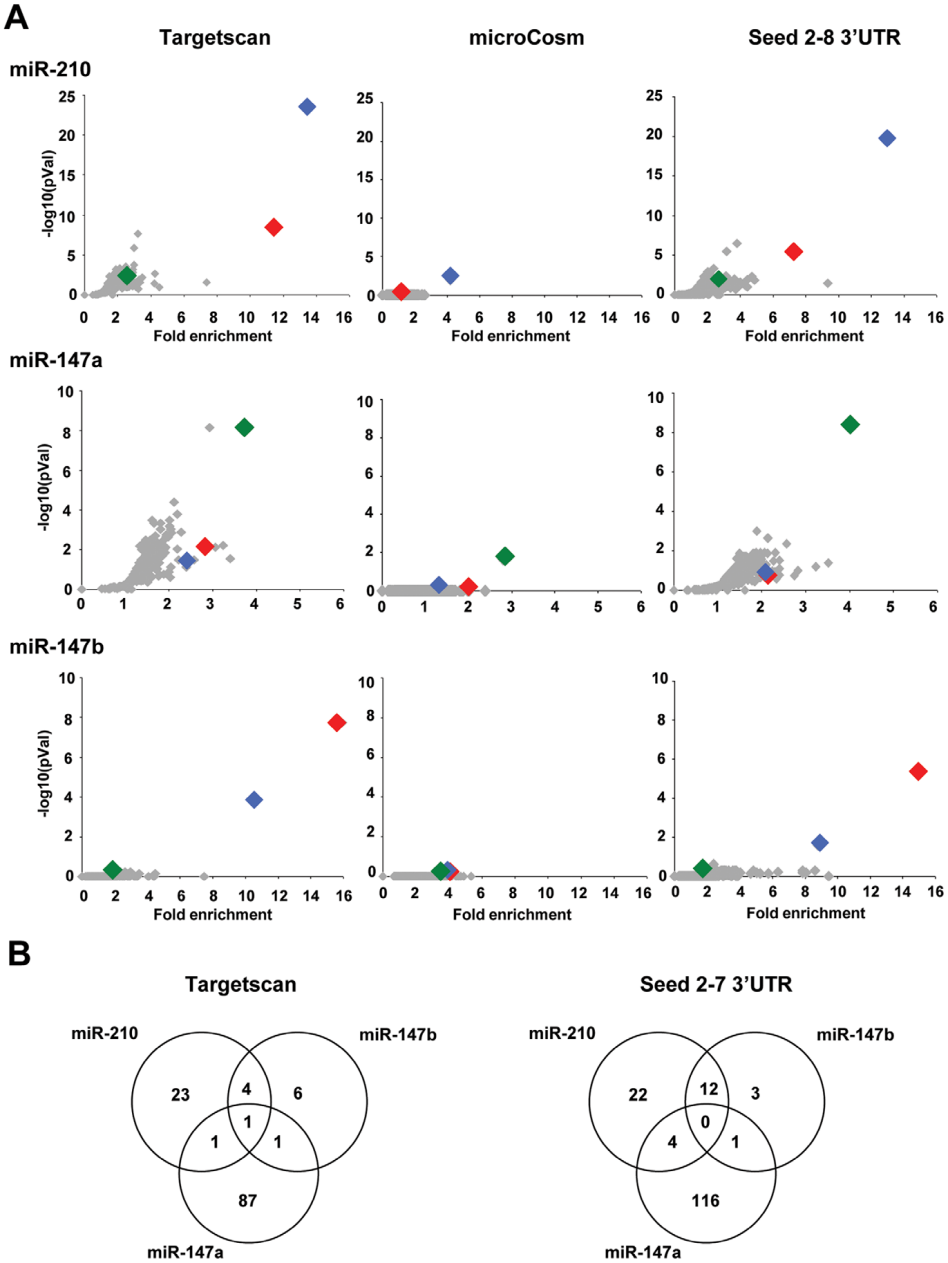
Enrichment of miR-210 and miR-147 family members-predicted targets in the different subsets of modulated transcripts following transfection by each miRNA candidate. A ) Graphs adapted from our webtool miRontop [33] showing the significance of the enrichment (represented as –log10 (adjPVal) according to the fold enrichment in experiments of overexpression of miR-210, miR-147a and miR-147b. Enrichment was calculated according to TargetScan, microCosm or a 2–8 seed search in 3′UTR. On each panel, miR-210, miR-147a and miR-147b are highlighted as blue, green and red dots, respectively. **B**) Venn diagram summarizing the predicted common targets of miR-210 and miR-147 family members using the combination of transcriptomic data and different bioinformatics prediction tools: TargetScan and a search of a 6 nt complementary seed 2–7 sequences in 3′UTR. Cut offs used for the selection of down-regulated transcripts: log2Average>8; logFC<-0.5 and Adj.pVal<0.05.

We then compared experimentally the influence of transfection of these 3 pre-miRNAs to a negative control pre-miRNA (pre-miR-Neg) on transcript levels in cultured cancer cells with human pan genomic arrays [36]. Studies were performed on lung Non Small Cell Lung Cancer adenocarcinoma A549 cells using methods recently described in details [25], [26], [37]. Shown are data collected from 3 independent biological experiments, RNA samples being harvested 48 hours after transfection (Figure 2). Statistical analysis revealed that a total of 1123 transcripts were significantly modulated in at least one of the experimental conditions when compared to the control condition (p<0.05, corresponding to 719, 120 and 434 genes for miR-147a, miR-147b and miR-210, respectively). As anticipated, ectopic expression of these 3 synthetic pre-miRNAs led to a large down-regulation of transcripts (Figure 2A). A functional annotation of the different signature patterns with the Ingenuity Pathway™ software was then performed. While there was an important overlap for “Molecular functions” terms such as “Cell Death”, “Cellular Growth and Proliferation” or “Cell Cycle” between the three conditions (Table S2), we found some “canonical pathways” specific to one or the other experimental conditions (Table S3). As previously shown for miR-210, we also found a close association of miR-147a and miR-147b signatures with “Oxidative phosphorylation”, “Mitochondrial dysfunction” and “Death Receptor signalling” [26]. Some highly specific pathways could be associated with miR-147a, the most significant being associated with cell cycle and DNA repair (“Mismatch Repair in Eukaryotes”; “ATM Signaling”; “Role of CHK Proteins in Cell Cycle Checkpoint Control”). Interestingly, all the pathways significantly modulated by miR-147b were also altered by miR-210. As shown in Figure 2B and 2C, the similarity between miR-210 and miR-147b-mediated transcriptomic changes was particularly underlined while most of miR-147a-mediated changes were specific. In order to identify the set of mRNA specifically targeted by each of these 3 miRNAs, we then looked at target enrichment in the population of down-regulated transcripts. Figure 3A shows the outputs of a bioinformatics analysis using our web tool “MiRonTop” [33], allowing the detection of miRNAs that significantly affect gene expression at a large scale. As expected, they indicate a specific over-representation of predicted targets in the set of downregulated transcripts for each of the 3 transfected miRNAs, using 3 different prediction tools, TargetScan, microcosm and a direct search of a 7 nt “seed” match in the 3′UTR (Figure 3A). Importantly, we found a significant co-enrichment of miR-210 and miR-147b predicted targets using these tools in cells transfected by either of these 2 miRNAs while no significant link was found with miR-147a (Figure 3A and Figure S1B). For instance, using TargetScan as prediction tool, we found an enrichment of more than 10 fold for miR-147b predicted targets in miR-210-transfected cells and similar data were found in the opposite conditions (Figure 3A, list of predicted targets in Table S4). We observed variations for this enrichment values when different zones of the transcripts were analyzed, with a maximum in the 3′UTR around the stop codon and low scores in the coding region (Figure S2). Interestingly, the co-enrichment observed between miR-210 and miR-147b was also detected when we considered conserved or non-conserved targets (Figure S3). Taken together, these data indicated that mRNAs that are knocked down following transfection by miR-210 and miR-147b tend to overlap substantially (between 40 and 70% depending on the prediction tool) but are different from those down-regulated by miR-147a (Figure 3A and Figure S1B). This picture appears quite different from the one drawn initially using only targets prediction algorithms that gave a strong overlap between all 3 miRNA targets (compare Figure 1B and Figure 3B). Overall, these results underscore the highly similar impact on transcriptome of miR-210 and miR-147b overexpression.

### Target Validation

**Figure 4:**
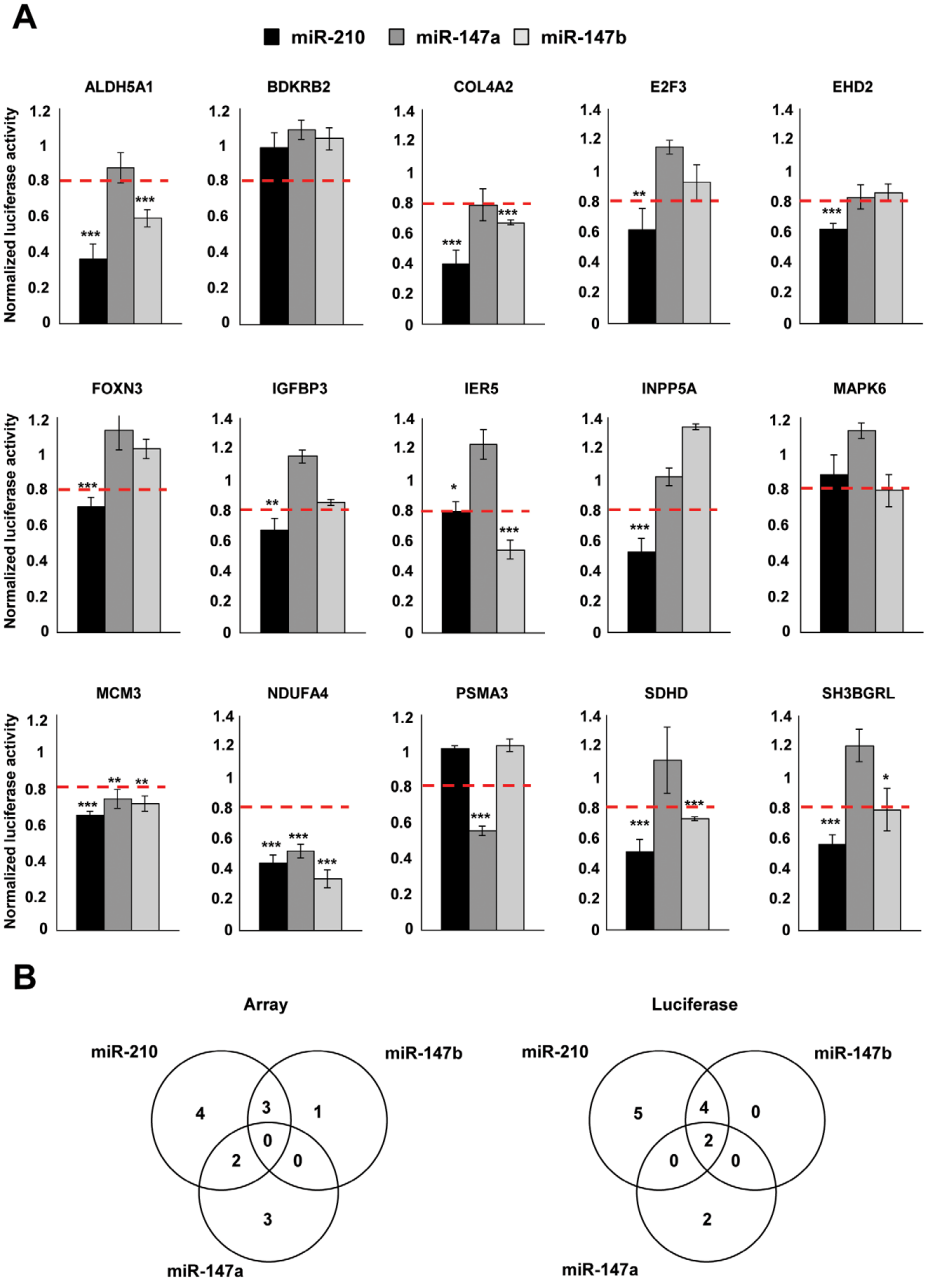
Targets validation using luciferase assays. A) Direct targeting of the 15 candidate transcripts by miR-210, miR-147a and miR-47b was analyzed *in vitro*. A549 cells were co-transfected with pre-miR-210, pre-miR-147a, pre-miR-147b or pre-miR-Neg and different pSI-Check-2™ constructs containing the 3′UTR of interest described in Table S5. Cells were harvested two days after transfection and luciferase activities were analyzed. B) Venn diagram summarizing the predicted (using the microarray approach) and validated (after luciferase assay) common targets of miR-210 and miR-147 family members. (*p<0.05, **p<0.005, ***p<0.0005).

For targets validation, we selected a set of genes among the list of transcripts significantly down-regulated in at least one experimental condition (Table S4). This validation set was based on different criteria, including the best p-values associated to transcripts modulation, the selection of transcripts that belong to the various categories visualized in the Venn diagram of Figure 3B and to annotations for gene ontology terms enriched in each experimental condition (provided by our bioinformatics tool miRontop): such as “mitochondria” and “apoptosis” for miR-210 and miR-147b and “cell cycle”for miR-147a. A subset of 15 transcripts was selected (Table 1). Based on the different alignments proposed by several target prediction algorithms, we then fused part of the 3′UTR of these transcripts (around 400 nt in length) to a luciferase reporter using the pSICheck-2 vector. The constructs, corresponding to the 3′UTR of NDUFA4, SDHD and E2F3, 3 validated targets of miR-210 have been described elsewhere [26]. Data showing the normalized luciferase activity following co-transfection of the pSICheck-2 plasmid with the different pre-miRNAs are presented in Figure 4A. Twelve out of 17 new predictions could be effectively confirmed, using a threshold of inhibition of 20% (Table 2). Interestingly, some transcripts that were slightly repressed in microarrays experiments were significantly inhibited in luciferase experiments: ALDH5A, SDHD and SH3BGRL, identified initially as miR-210-only targets turned out to be also targeted by miR-147b while the opposite situation occurred with IER5. Finally, MCM3, a miR-147a-only predicted target was found to be equally inhibited by the 3 miRNAs. Overall, the global picture summarizing the target specificity for these 15 transcripts appeared quite similar for microarray and luciferase assays and underscored the close relationships between miR-210 and miR-147b (Figure 4B).

**Table 1:** List of the 15 trancripts selected for validation.

Name	Accession	Function	miR-210(M)	miR-147a(M)	miR-147b(M)	miR-210(seed)	miR-147a(seed)	miR-147b(seed)
ALDH5A1	NM_001080.3	dehydrogenase (M)	**−0.67**	0.08	−0.45	2–8	1–8	2–7
BDKRB2	NM_000623.2	Bradykinin Receptor	**−0.58**	−0.31	−0.35	1–9	(6)	2–9
COL4A2	NM_001846.2	Collagene	**−1.51**	−0.05	−**0.96**	2–7	1–9	1–9
E2F3*	NM_001949.2	Transcription factor	0.07	−0.25	0.05	1–8	(4)	2–7
EHD2	NM_014601.2	Actin biding protein	**−0.87**	**−0.86**	−0.41	2–10	1–8	1–7
FOXN3	NM_005197.3	Transcription factor	**−0.55**	−0.02	−0.32	2–14	(19)	1–7
IER5	NM_016545.4		−0.33	−0.37	**−0.64**	1–7	(0)	2–15
IGFBP3	NM_000598.4	Insulin binding protein	**−1.32**	−0.34	−**0.95**	1–11	1–9	2–7
INPP5A	NM_005539.3	Phosphatase	**−0.75**	**−0.60**	−0.28	1–8	1–9; 1–7	2–7
MAPK6	NM_002748.3	Ser/Thr kinase	0.35	**−0.65**	0.24	(0)	2–7	(0)
MCM3	NM_002388.3	pre-replication complex	0.02	**−0.92**	−0.13	(0)	2–8	(0)
NDUFA4*	NM_002489.2	dehydrogenase (M)	**−1.75**	**−2.17**	**−2.15**	2–11	(0)	2–9
PSMA4	NM_002789.4	proteasome	−0.25	**−1.05**	0.02	(0)	1–10	(0)
SDHD*	NM_003002.1	dehydrogenase (M)	−0.45	0.00	−0.31	2–7	(0)	2–13
SH3BGRL	NM_003022.1	unknown	**−0.91**	0.27	−0.34	2–12	(0)	2–7

The list of 15 transcripts predicted to be targeted at least by one of the 3 miRNAs using the bioinformatics tool miRonTop are listed. M correspond to the Logarithm (base 2) of the ratio between each miRNA versus miR Neg. Bold values indicated a ratio above the threshold selected. The different types of base-pairing (seed) are listed; numbers inside brackets indicate the number of potential miRNA sites. * corresponds to previously validated targets from [26], [50].

**Table 2:** Table summarizing the data of microarrays and luciferase assays concerning the subset of 15 transcripts.

	miR-210		miR-147a		miR-147b	
	MiRonTop prediction	Luciferase assay	MiRonTop prediction	Luciferase assay	MiRonTop prediction	Luciferase assay
ALDH5A1	X	X				X
BDKRB2	X					
COL4A2	X	X		X	X	X
E2F3		X*				
EHD2	X	X	X			
F0XN3	X	X				
IER5		X			X	X
IGFBP3	X	X			X	
INPP5A	X	X	X			
MAPK6			X			
MCM3		X	X	X		X
PSMA4			X	X		
NDUFA4	X	X*		X	X	X
SDHD	X	X*				X
SH3BGRL	X	X				X

* corresponds to previously validated targets from [26], [50].

### Cellular Phenotypes Triggered by miR-147a, miR-147b and miR-210 Overexpression

**Figure 5:**
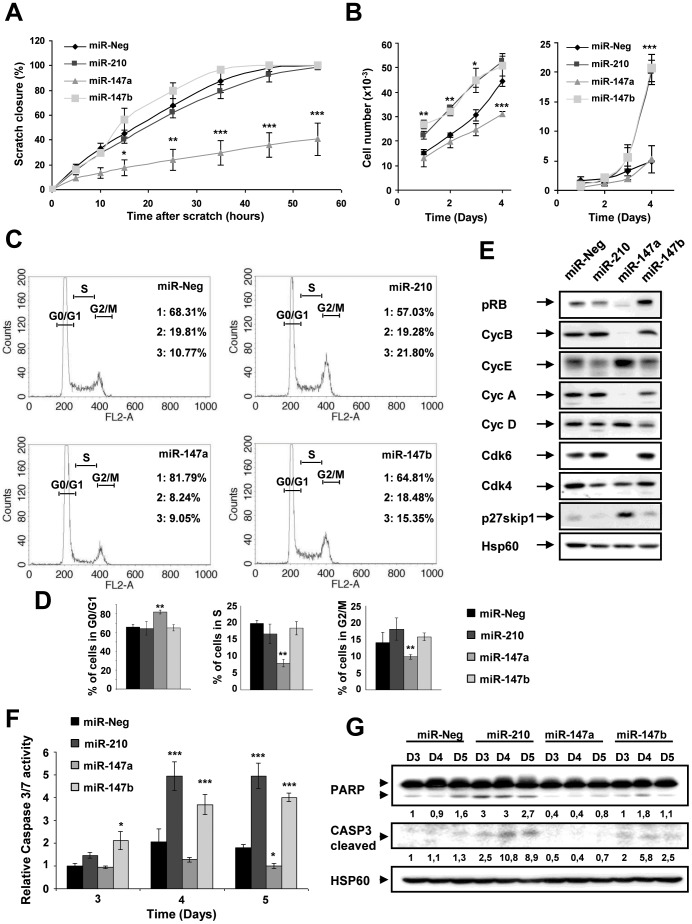
Biological consequences of miR-210, miR-147a and miR-147b overexpression on A549 cells proliferation and viability. A549 cells were transfected with 10 nM pre-miR-210, pre-miR-147a, pre-miR-147b or pre-miR-Neg and analyzed for several proliferation (A-E) and viability (F-G) parameters. A) Confluent cell monolayer was wound and filmed for 55h under light time laps microscope. Curves represent wound beds closure quantified by measuring the wound bed surface at the indicated times after injury using the Image J software. Values are expressed in percentage of the initial surface and correspond to the mean ± SD of 3 microscope fields. B) Effect of miR-210 and miR-147 family on A549 cell proliferation. Exponentially growing A549 cells were transfected and counted each day during 4 days with blue Trypan. Data show mean ± SD values of trypan blue negative (left panel) and trypan-blue positive cell number (right panel) from 2 independent experiments performed in triplicate. C) Cells were stained with propidium iodide and analyzed by flow cytometry. The G0/G1 (1), S (2) and G2/M (3) fractions were quantified in each condition. D) Quantification of each of these 3 fractions (G0/G1, S and G2/M) from 3 independent experiments. E) Expression of Cyclin D, Cyclin A, Cyclin E, CDK4, CDK6, pRB (6 molecules involved in G1 phase progression), p27Kip1 (inhibitor of G1 phase progression) and Cyclin B (involved in G2/M phase) were assessed by Western blot. Hsp60 corresponds to the loading control. F) Caspase 3/7 assay was performed at 3, 4 and 5 days after transfection. Data are mean ± SD values of 3 independent experiments performed in triplicate. See also Figure S4A. G) Expression of active caspase-3 (cleaved) and PARP, a substrate of caspase-3 was analyzed by Western blot. HSP60 corresponds to the loading control. Normalized densitometric quantification are shown for each lane. See also Figure S4C and Figure S4D (*p<0.05, **p<0.005, ***p<0.0005).

We next investigated the effects of these three miRNAs on several cellular parameters. We first analyzed their effects in a scratch wound repair assay on a collagen type I substrate (Figure 5A). A strong wound closure delay was observed following pre-miR-147a transfection while no significant effect could be detected with pre-miR-210 and pre-miR-147b. A significant inhibition of cell proliferation by miR-147a was also observed in regular condition of growth on plastic (Figure 5B, left panel) while an opposite early transient stimulation by both miR-147b and miR-210 was noticed. However, a late toxic effect of these 2 miRNAs was detected 4 days of transfection, as evidenced by a significant increase of trypan blue positive cells (Figure 5B, right panel). Cell cycle analysis was then performed and indicated that miR-147a mediated cell cycle arrest in G1 phase while miR-210, and to a lesser extent miR-147b slightly increased the fraction of cells in the S/G2 phase (Figure 5C and 5D). The strong inhibitory action of miR-147a on cell proliferation was underlined by the down-regulation of pRB, cyclins (A and B) and cyclin-dependent kinase 6 (Cdk6) as well as a massive induction of the cyclin-dependent kinase inhibitor 1B (p27 Kip1) (Figure 5E).

In order to confirm the effect of miR-210 and miR-147b on cell death observed 4 days after transfection, we next measured caspase 3/7 activity, which revealed significant induction of apoptosis in A549 cells by miR-210, as previously demonstrated [26] but also by miR-147b (Figure 5F). This effect was further confirmed by the specific detection of the cleaved forms of Poly ADP ribose polymerase (PARP) and caspase-3 as well as a decrease of pro-caspase-3 in cell lysates of miR-210 and miR-147b-transfected A549 cells (Figure 5G and S4C-D). Therefore, these experiments underlined the close proximity of miR-210 and miR-147b regarding apoptotic-mediated cellular effects while miR-147a appears as a potent inhibitor of cell proliferation and migration.

### Distinct Regulation of miR-210 and miR-147 Family Members in A549 Cells

**Figure 6:**
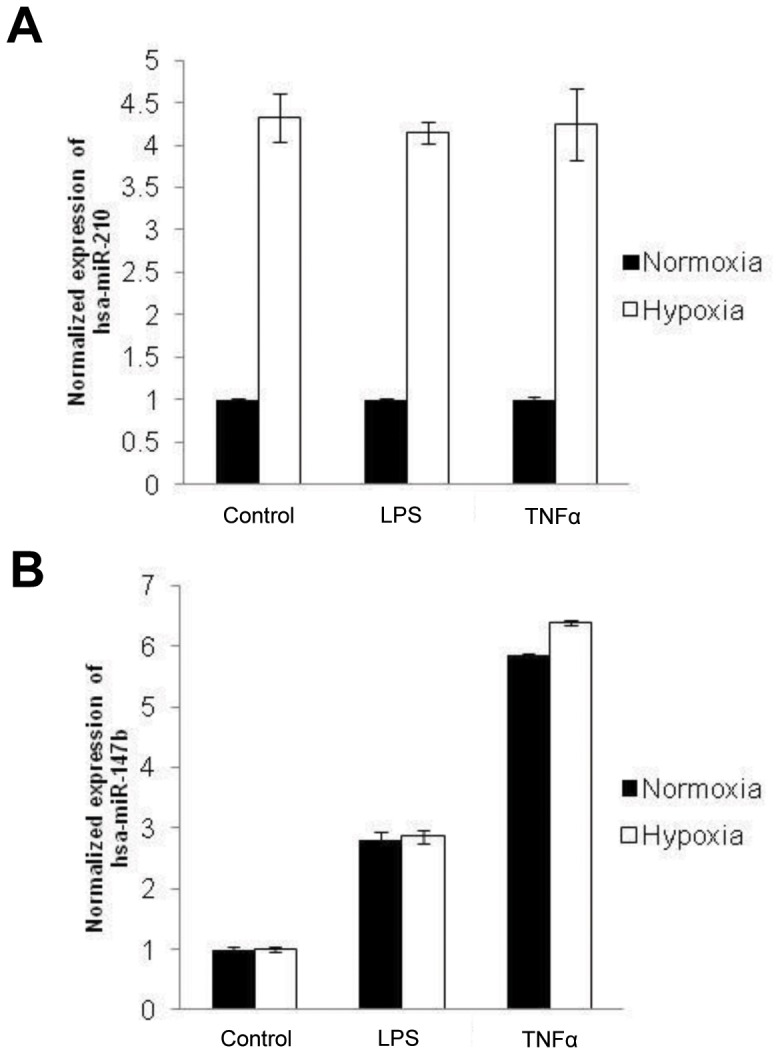
miR-147b and miR-210 expression in response to LPS, TNFα and hypoxia. MiR-210 (A) and miR-147b (B) expression was monitored by qPCR on RNA from A549 cells stimulated with 10 µg/ml LPS or 10 ng/ml TNFα with or without exposure to 1% O2 for 48 hours. Mean ± SEM is representative of 3 independent experiments carried out in triplicate**.**

While it is well known that miR-210 expression is under the control of Hypoxia-Inducible Factor-1 alpha (HIF-1α) in virtually all cell lines tested, including A549 cells, the regulation of expression of hsa-miR-147a and hsa-miR-147b has not been well documented yet. Based on a previous report describing the regulation of mmu-miR-147 (*ie:* the homolog of hsa-miR-147b) in murine macrophages upon Toll-like receptor stimulation [38], we treated A549 cells with LPS and TNFα in normoxic or hypoxic conditions and measured the levels of the 3 mature miRNAs using TaqMan qRT-PCR. As expected, miR-210 was significantly induced in A549 cells under hypoxic condition but was insensitive to LPS or TNFα treatment (Figure 6A). In contrast, a strong induction of miR-147b by TNFα and to a lesser extent by LPS was observed (Figure 6B), while no significant stimulation of miR-147a could be detected in these conditions (data not shown).

## Discussion

The ability of miRNAs to interact with many targets and the possibility for some of them to share a same target increase tremendously the complexity of biological networks. In that context, the use of bioinformatics tools that explore miRNA influence on datasets [30], [31], [32], [33] is mandatory to establish a minimal set of mRNA modulated by a given miRNA and offer the opportunity to identify miRNAs with overlapping regulatory functions. Using our recently described application MiRonTop [33] and a large set of miRNA-overexpressed microarray experiments, we noticed indeed a close relationship between two miRNAs from distinct families, hsa-miR-147b and hsa-miR-210. While these 2 species only share the same “minimal” seed sequence, hsa-miR-147b has a homolog, hsa-miR-147a, which differs only with a 1 nt substitution in the seed sequence. We could first show the strong similarity between miR-210 and miR-147b at a whole genome transcriptome level while it appeared that miR-147a-mediated changes were highly specific. Second, we could validate a significant number of common predicted targets of miR-210 and miR-147b using luciferase assay. Third, we could confirm this close proximity at a functional level on several cellular parameters.

The methodology used in our study was based on the overexpression of these different miRNAs, which might induce some drawbacks, because the high expression of synthetic miRNAs does not reflect a physiological induction of these miRNAs. This is not the case here, since a concentration as low as 0.1 nM of miR-210 or miR-147b mimics was still able to induce a significant apoptotic effect (Figure S4). Our more recent experiments using a lentiviral approach confirmed that expression of miR-210 within ∼2fold of physiological induction by hypoxia also resulted in a significant co-enrichment of miR-210 and miR-147b predicted targets (data not shown). It will be informative to further confirm these data through the knock-down of the endogenous miRNAs. However, this specific approach appears quite complex in our model. Indeed, hypoxia and TLR stimulation, which increase miR-210 and miR-147b, respectively, induce very large and different transcriptional programs that will likely interfere with an efficient comparison of the corresponding data sets and also mask the more subtle effects of these miRNAs on their mRNA targets.

These data provide an interesting and natural example illustrating the seed-dependent function of miRNAs. The importance of the seed has been widely explored through hundred of examples of miRNAs – transcripts pairs [7]. While the miR-210/miR-147b proximity shown in our study underscores this key molecular rule, it also provides several original findings. First, this represents to our knowledge one of the first example of a functional similarity between miRNAs species based on a 6 nt “minimal seed only” identity. Similar studies have focused on the mRNA targeting overlap among homologous miRNAs, in particular the miR-15/107 group, composed of ten distinct mature miRNAs [39]. Using a similar approach, these authors have shown that transfection of several members of this group gave nearly identical expression profiles and similar cellular phenotypes [39], [40], [41]. They concluded that these miRNAs are members of a single miRNA group, whose membership could be defined on sequence identity in the mature miRNA 5′end, that included a 6 nt sequence [39]. It appears noteworthy however that most of these sequences also shared some identity in either the 8^th^ nucleotide of the seed or in the 3′ end section. Second, the 1 nt substitution observed between miR-147a and miR-147b, located in the seed at the 5^th^ base, corresponds to a change of U (in miR-147a) into a C (in miR-147b), indicating that all “seed-dependent” miR-147b targets would tolerate a GU wobble, as it has often been shown [6]. Herein contradiction with this model, a very small percentage of miR-147b targets were also down-regulated by miR-147a in the microarray data. Moreover, luciferase validation experiments demonstrated that only 1 target out of 7 miR-147b targets was significantly down-regulated by miR-147a, NDUFA4, with an extensive 7 nt pairing to the 3′ portion of the miRNAs that could compensate for this single mismatch in the seed, as previously proposed for the let-7 site in lin-41 transcript [7]. Overall, our data strongly support that a minimal 6 nt seed sequence only found in miR-210 and miR-147b represents the major functional component of these 2 miRNAs.

On a more general view, it appears instrumental to address whether a similar situation could be observed in other miRNAs with degenerate seed sequences. Bioinformatics analysis of human miRNA sequences from miRBase (release 16) indicated that 207 seed sequences out of 872 are shared by at least two miRNAs (Figure S5 and Table S5). Interestingly, only a minority of these sequences are classified into identical families (*ie*: miRBase families) based on the overall sequences alignments from non-coding RNAs [42]. For instance, in the case of a seed shared by 2 miRNAs (Figure S5, “2 miR/seed” histogram), we found a total of 136 shared seeds including only 47 seeds belonging to miRNAs from the same family (34,6%). Overall, this observation may indicate that multiple other miRNAs are indeed in the same situation as the one described here for miR-210 and miR-147b and would need to be further clarified in terms of putative functional redundancy.

The close relationship observed for miR-210 and miR-147b in microarrays experiments was confirmed at the cellular level using several assays. We show that both these miRNAs induced a delayed apoptosis, as evidenced by trypan blue staining, caspase-3 activity and PARP cleavage (Figure 5), confirming the pro-apoptotic effect of miR-210 in A549 cells [26]. This effect could be likely mediated by the targeting of several mitochondrial components by these 2 miRNAs, such as ISCU, NDUFA4 or SDHD, inducing mitochondrial dysfunctions associated with induction of reactive oxygen species, decrease of mitochondrial membrane potential and caspase-3 activation [26], [43], [44]. By contrast, the impact of miR-147a was mainly at the level of cell division, since miR-147a overexpression led to a highly disproportionate number of modulated cell cycle genes (p<10^−15^, Table S1). Several canonical pathways associated to cell cycle (control of chromosomal replication, G_1_/S checkpoint regulation, G_2_/M DNA damage checkpoint regulation) were also significantly altered in miR-147a-transfected cells (Table S2). In agreement with such a signature, miR-147a transfection led to a strong inhibitory action on cell proliferation and a G_0_/G_1_ arrest in a pRB-dependent mechanism. Several cell-cycle-related genes, such as CDK6 or MCM3, represent putative miR-147a targets. MCM3 was indeed validated as a *bona fid*e miR-147a target by luciferase assay, but additional targets are probably involved in the observed phenotype. An upregulation of miR-147a has been reported in several cancers [45], [46]. However, we were unable to detect a significant expression of this miRNA in our small RNA Seq database. This is consistent with its current “not evidence-based experimental” status in miRBase [47]. It remains that the human genome, as well as other primate sequences (*Pan troglodytes, Pongo pygmaeus* and *Macaca mulatta*) contain a predicted precursor hairpin for miR-147a. The situation is different for miR-210 and miR-147b. MiR-210 locus is located on chromosome 11 and has been classified as an “intergenic” miRNA processed from an uncharacterized pri-miRNA transcript. Multiple studies have consistently established that miR-210 induction is a feature of the hypoxic response in both normal and transformed cells and its overexpression has been detected in a variety of cardiovascular diseases and solid tumors (for review, [48]). A wide spectrum of miR-210 targets have been identified, with roles in mitochondrial metabolism, angiogenesis, DNA repair, and cell survival. MiR-147b is located in exon 4 of the normal mucosa esophagus specific 1 (NMES1) transcript, downstream of the NMES1-coding sequence, and possesses the same mature sequence as mmu-miR-147, with a similar location on the mouse homologous gene. As shown in Figure S6, using data from our “small RNA Seq database”, miR-147b is indeed significantly expressed in three distinct human tissues or cell line, including A549 cells. Moreover, these reads are totally consistent with a transcription from the NMES1 locus. Expression of mmu-miR-147 has been shown to be induced upon Toll-like receptor (TLR) stimulation in murine macrophages and has been linked to the sensitivity of the inflammatory response, suggesting that it could be part of a negative feed-back loop of the TLR pathway [38]. We have confirmed these data in human epithelial cells using TaqMan qRT-PCR, showing a significant upregulation of miR-147b by both LPS and TNFα.

Our data interestingly connect the molecular pathways regulated by miR-210 and miR-147b, suggesting that they could drive a common stress-related response following either hypoxia or inflammation. This suggests that these two different external stimuli could lead to a similar intracellular molecular adaptation regarding metabolic switch as suggested by several reports about miR-210 function [48]. It will be of particular interest to study the expression and function of these two miRNAs in ischemic and inflammatory diseases as well as in pathological settings where both hypoxia and inflammation co-exists such as cancer.

Overall, our study highlights the strong functional divergences that can be observed between miRNAs sharing an overall sequence similarity and shed some new light on the approaches that should be developed to improve the classification of miRNAs. This could be extremely beneficial to all studies where complex miRNA molecular networks are dissected. More specifically, our study is the first demonstration of the strong functional link existing between miR-147b, associated within inflammatory response, and miR-210 associated with hypoxia and cancer, *i.e.* two miRNAs previously associated to distinct regulatory pathways and cellular role.

## Materials and Methods

### Cell Culture

The lung adenocarcinoma A549 cell line was purchased from the American Type Culture Collection and was grown in DMEM supplemented with L-glutamine (8 mM), 10% FBS, Penicillin (50 U/ml) and Streptomycin (50 µg/ml) at 37°C in a humidified 5% CO_2_ air atmosphere. A Bug-Box™ anaerobic workstation (Ruskinn Technology) set at 1% oxygen, 94% nitrogen and 5% carbon dioxide was used for hypoxic conditions.

### Transfection

Chemically synthesized miRNA duplexes pre-miR-210, pre-miR-147a and pre-miR-147b and control pre-miRNA (pre-miR-Neg # 1) were purchased from Ambion. A549 cells were transfected at 50% confluency with Lipofectamin RNAi MAX™ (Invitrogen) and with pre-miRNAs at a final concentration of 10 nM.

### RNA Extraction

Cells were homogenized in 2 ml of TRIzol reagent (Invitrogen). Total RNAs including small RNAs were extracted using the miRNeasy kit (Qiagen) according to the manufacturer’s instructions. RNA quality was checked using the bioanalyzer 2100 (Agilent Technologies). RNA concentration was determined using the ND-1000 micro-spectrophotometer (NanoDrop Technologies).

### Quantitative RT-PCR of Mature miRNA

Mature miRNA expression was evaluated using TaqMan MicroRNA Assays (Applied Biosystems) and the Lightcycler 480 detection system (Roche). All reactions were performed in duplicate. Expression levels were normalized to RNU6B and calculated using the comparative C_T_ method (2^−ΔΔCT^).

### Microarrays

Pangenomic microarrays were printed using the human RNG/MRC oligonucleotide collection as previously described [36]. RNA were labelled and hybridized as previously described [26]. Three biological replicates were performed for each comparison. The experimental data and microarray design have been deposited in the NCBI Gene Expression Omnibus (GEO) (http://www.ncbi.nlm.nih.gov/geo/) under serie GSE33247.

#### Statistical analysis

Normalization was performed using the Limma package available from Bioconductor (http://www.bioconductor.org). Intra slide and inter slide normalization was performed using the Print Tip Loess and the quantile methods, respectively. Means of ratios from all comparisons were calculated and B test analysis was performed. Differentially expressed genes were selected using Benjamini-Hochberg correction of the *p*-value for multiple tests, based on a *p*-value below 0.05 and a fold change cut off (logRatio >0.5).

#### Biological Theme Analysis

Data from expression microarrays were analyzed for enrichment in biological themes (Gene Ontology molecular function and biological process) and build biological networks built using Ingenuity Pathway Analysis software (http://www.ingenuity.com/) and Mediante (http://www.microarray.fr:8080/merge/index) [49], an information system containing diverse information about our probes and data sets.

#### Targets analysis

MiRonTop [33] is an online java web tool (available at http://www.microarray.fr:8080/miRonTop/index) that integrates DNA microarrays data to identify the potential implication of miRNAs on a specific biological system. Briefly, MiRonTop ranks the transcripts into 2 categories (‘Upregulated’ and ‘Down regulated’), according to thresholds for expression level and for differential expression. It then calculates the number of predicted targets for each miRNA, according to the prediction software selected (Targetscan, microCosm, PicTar, exact seed search: 2–7 or 1–8 first nucleotides of the miRNA, TarBase v1), in each set of genes. Enrichment in miRNA targets in each category is then tested using the hypergeometric function.

### Molecular Constructs

3′UTR sequences from *ALDH5A1, BDKRB2, COL4A2, EHD2, FOXN3, IER5, IGFBP3, INPP5A, MAPK6, MCM3, PSMA4* and *SH3BGRL*, were cloned in the pSI-CHECK™-2 vector (Promega) downstream of the *Renilla* luciferase using *Xho*I and *Not*I restrictions sites. Constructs for *E2F3, NDUFA4* and *SDHD* have been previously described [26]. The sequences of primers used for each construct are described in Table S1.

### miRNA Targets Validation by Luciferase Assay

A549 cells were plated in 96-well plates and transfected with 200 ng of pSI-CHECK™-2 constructs and 10 nM of pre-miRNAs using Lipofectamine 2000 (Invitrogen). The medium was replaced 8 hours after transfection with fresh medium containing 10% FCS, L-glutamine and supplemented with penicillin and streptomycin. 48 hours after transfection, firefly and *Renilla* Luciferase activities were measured using the Dual-Glo™ Luciferase assay (Promega).

### Cell Cycle Analysis

A549 cells were seeded in 12 well plates, transfected 24 hours later and trypsinized 72 hours after transfection for cell cycle analysis. Cells were collected by centrifugation at 1500 rpm for 5 min, fixed with cold ethanol 70% and resuspended in PBS containing 1% NP40, propidium iodide (20 µg/ml) and RNAseA (20 µg/ml). After 15 mn cells were analyzed by flow cytometry using FACSCALIBUR (Becton Dickinson).

### Caspase 3/7 Assay

The activation of executioner caspase-3 and -7 in A549 cells was determined using the Caspase-Glo 3/7 Assay kit (Promega) according to the manufacturer’s instructions. A549 cells were plated in triplicate in 96-well plates and transfected as described above. Luminescence was quantified after 1 hour of incubation with the caspase substrate on a luminometer.

### Western Blot Analysis

Cells were lysed in Laemmli buffer and the protein concentration determined using the Bradford assay (Biorad). 40 µg of proteins were resolved by SDS-PAGE and transferred onto a PVDF membrane (Millipore). Membranes were blocked in 5% non-fat milk in TN buffer (50 mM Tris-HCl pH 7.4, 150 mM NaCl) and incubated in the presence of the primary and then secondary antibodies. After washing in TN buffer containing 1% Triton-X100, immunoreactive bands were visualized with the ECL system (Amersham Biosciences). Polyclonal or monoclonal antibodies to CCNA (C19), p27Kip1 (C19), CDK4 (C22), CDK6 (C21), CCNB1 (GNS1), CCND1, CCNE1 and HSP60 (K19) were purchased from Santa Cruz Biotechnology. Anti-RB (MAB3186) mouse mAb and anti-caspase-3 (total and cleaved) were from Millipore and Cell Signaling, respectively.

### *In vitro* Wound Scratching Assay

Confluent A549 cells were wounded using pipet tips and wound bed closure was recorded by videomicroscopy for 48 hours on an Axiovert 200 M inverted microscope (Carl Zeiss) equipped with 37°C and 5% CO2 regulated insert (Pecon GmbH, Germany). Brightfied images were taken each hour through a 10*6* phase contrast objective with a CoolSNAPHQ CCD Camera managed by Metamorph Software (Roper Scientific, Evry, France). Wound bed areas were quantified using the NIH ImageJ sotware (http://rsb.info.nih.gov/ij/).

### Statistical Analysis

Results are given as the mean ± S.E.M. Statistical analysis was performed using the Student’s t-test as provided by Microsoft Excel™ and the null hypothesis was rejected at the 0.05 level (*p<0.05, **p<0.005, ***p<0.0005).

## Supporting Information

Figure S1 Overlap between predicted targets of miR-210 and miR-147 family members. A) *In silico* evaluation of the common predicted targets between hsa-miR-210, hsa-miR-147a and hsa-miR-147b using TargetScan or microCosm. B) Overlap between the predicted targets for each of the 3 miRNAs that are significantly down-regulated following transfection by each of the miRNA candidates. Data were calculated using our webtool miRontop (Lebrigand et al. 2010, Bioinformatics) using the following cut offs : log2Average>8; logFC<-0,5 and Adj.pVal<0,05. Note that an important percentage of genes knocked down by miR-210 were also knocked down by miR-147b but not by miR-147a.
(TIF)

Figure S2 Graphs adapted from our webtool miRontop (Le Brigand et al. 2010, Bioinformatics) showing the significance of the enrichment (represented as –log10 (adjPVal) according to the fold enrichment in experiments of overexpression of hsa-miR-210, hsa-miR-147a and hsa-miR-147b. Enrichment was calculated according to a 2–7 seed search in distinct regions of the transcripts. On each panel, hsa-miR-210, hsa-miR-147a and hsa-miR-147b are highlighted as blue, green and red dots, respectively.
(TIF)

Figure S3 Graphs adapted from our webtool miRontop (Le Brigand et al. 2010, Bioinformatics) showing the significance of the enrichment (represented as –log10 (adjPVal) according to the fold enrichment in experiments of overexpression of hsa-miR-210, hsa-miR-147a and hsa-miR-147b. Enrichment was calculated according to a 1–8 seed search in 3′UTR or using the conserved or non-conserved miRNA targets prediction database from TargetScan. On each panel, hsa-miR-210, hsa-miR-147a and hsa-miR-147b are highlighted as blue, green and red dots, respectively.
(TIF)

Figure S4 Dose-response effect of miR-210, miR-147a and miR-147b on A459 cells viability. A549 cells were transfected with 10 nM, 1nM, 0,1nM or 0,01nM of hsa-pre-miR-210, hsa-pre-miR-147a, hsa-pre-miR-147b or pre-miR-Neg and analyzed for several viability parameters. A) Caspase 3/7 assay was performed at 3, 4 and 5 days after transfection. Data are mean ± SD values of 2 independent experiments performed in triplicate. B) Cells were collected 48 h after transfection and the relative miR-210 levels were determined using a TaqMan assay. C) Expression of pro-caspase-3 was analyzed by Western blot in A549 cells transfected with each indicated pre-miRNA at 10 nM. Hsp60 corresponds to the loading control. D) Densitometric quantification of pro-caspase-3 gene normalized for Hsp60 signal.
(TIF)

Figure S5 Usage of distinct seed (2–7 nt) sequences among human miRNAs. Pie-chart showing the representation of seed 2–7 among all human mature miRNAs. On the 872 distinct human seed sequences in miRBase v16, 665 are unique and 207 are shared by two or more miRNAs (miR). MiRNAs sharing the same seed sequence can belong to distinct miRBase families, thus the number of distinct miRBase families was reported for each shared seed sequence. The proportion of represented miRBase family in the shared seeds is shown as a barplot for the seeds shared by 2, 3 and 4 miRNAs.
(TIF)

Figure S6 Expression of hsa-miR-147b in different human tissues using Small RNA Seq. Screenshot of the human miR-147b locus generated by the UCSC genome browser (hg19 assembly). Black boxes correspond to coverage of each base position (bigwig files). Data from 3 human samples were loaded, with sample description on the left part of the tracks. Annotated transcripts of the locus, including miR-147b, are shown at the bottom (red box with arrows showing the strand direction). Total RNA were isolated from colon cancer, Non Small Cell Lung Cancer cell line A549 and normal airway epithelial cells (obtained from inferior turbinates from patients who underwent surgical intervention for nasal obstruction). The SOLiD™ Small RNA Expression Kit (Applied Biosystems, Life Technologies Corporation) was used to build a library of double-stranded DNA molecules from the population of small RNAs present in the different samples, which were then read using the Applied Biosystems SOLiD™ System sequencing according to the manufacturer’s instructions. Libraries were amplified by emulsion PCR and sequenced on SOLiD according to the manufacturer’s instructions. Read length was 35 bp. Color-space reads were matched against annotated databases using the Small RNA Analysis Pipeline Tool v5.0 (RNA2MAP), provided by Applied Biosystems, using the following parameters: one color-space mismatch within the first 18 bases of the reads, called the ‘seed sequence’ and two color-space mismatches on the following positions of the reads. Reads were matched against the human genome (hg19).
(TIF)

Table S1 List of forward and reverse primers used for cloning 3′UTR sequences in pSI-CHECKTM-2 (Promega) by cloning behind the *Renilla* luciferase in the *Xho*I and *Not*I restrictions sites.
(DOCX)

Table S2 List of themes corresponding to “Molecular Function” annotations identified by Ingenuity Pathway Analysis in response to overexpression of hsa-miR-210, hsa-mir-147a or hsa-miR-147b. The probability to obtain the number of genes in a certain pathway in the list of differentially expressed genes was compared with the representation of the same pathway among all the genes on the microarray and was calculated as a Fisher’s exact probability (p-value cut-off = 0.001).
(DOCX)

Table S3 List of themes corresponding to “canonical pathways” annotations identified by Ingenuity Pathway Analysis in response to overexpression of hsa-miR-210, hsa-miR-147a or hsa-miR-147b. The probability to obtain the number of genes in a certain pathway in the list of differentially expressed genes was compared with the representation of the same pathway among all the genes on the microarray and was calculated as a Fisher’s exact probability (p-value cut-off = 0.05). The genes modulated in each theme are represented.
(DOCX)

Table S4 Full list of the predicted targets transcripts down-regulated following hsa-miR-210, hsa-miR-147a or hsa-miR-147b overexpression in A549 cells. The transcripts predicted to be targets (seed 2–7 in 3′UTR) are highlited. Logarithm (base 2) of the average expression, logarithm (base 2) of the ratio of miR-210/miR-Neg and false discovery rate p-values using the Benjamini-Hochberg correction are represented. ID: correspond to RNG oligo IDs that give access to transcripts and probes annotations through our information system Mediante (http://www.microarray.fr:8080/merge/index).
(DOCX)

Table S5 Association of microRNAs and miRBase family with each human seed. In miRBase v16, 872 distinct seed sequences were found in human microRNA. For each seed, the number of microRNA having this seed is reported (count_mirna) along with the number and names of microRNA having this seed in the 5′ arm (count_mirna_5p, mirna_5p) or the 3′ arm (count_mirna_3p, mirna_3p). For each seed and each arm side, the number and names miRBase families are displayed (count_fam_5p, count_fam_3p, fam_5p, fam_3p). The total number of distinct miRBase families for each seed is also displayed (count_fam).
(XLSX)
