# Supplementary figures and images for: “Seed-Milarity” Confers to hsa-miR-210 and hsa-miR-147b Similar Functional Activity
Source: PLoS One. 2012 Sep 13;7(9):e44919. doi: 10.1371/journal.pone.0044919 (PMC3441733; doi:10.1371/journal.pone.0044919)

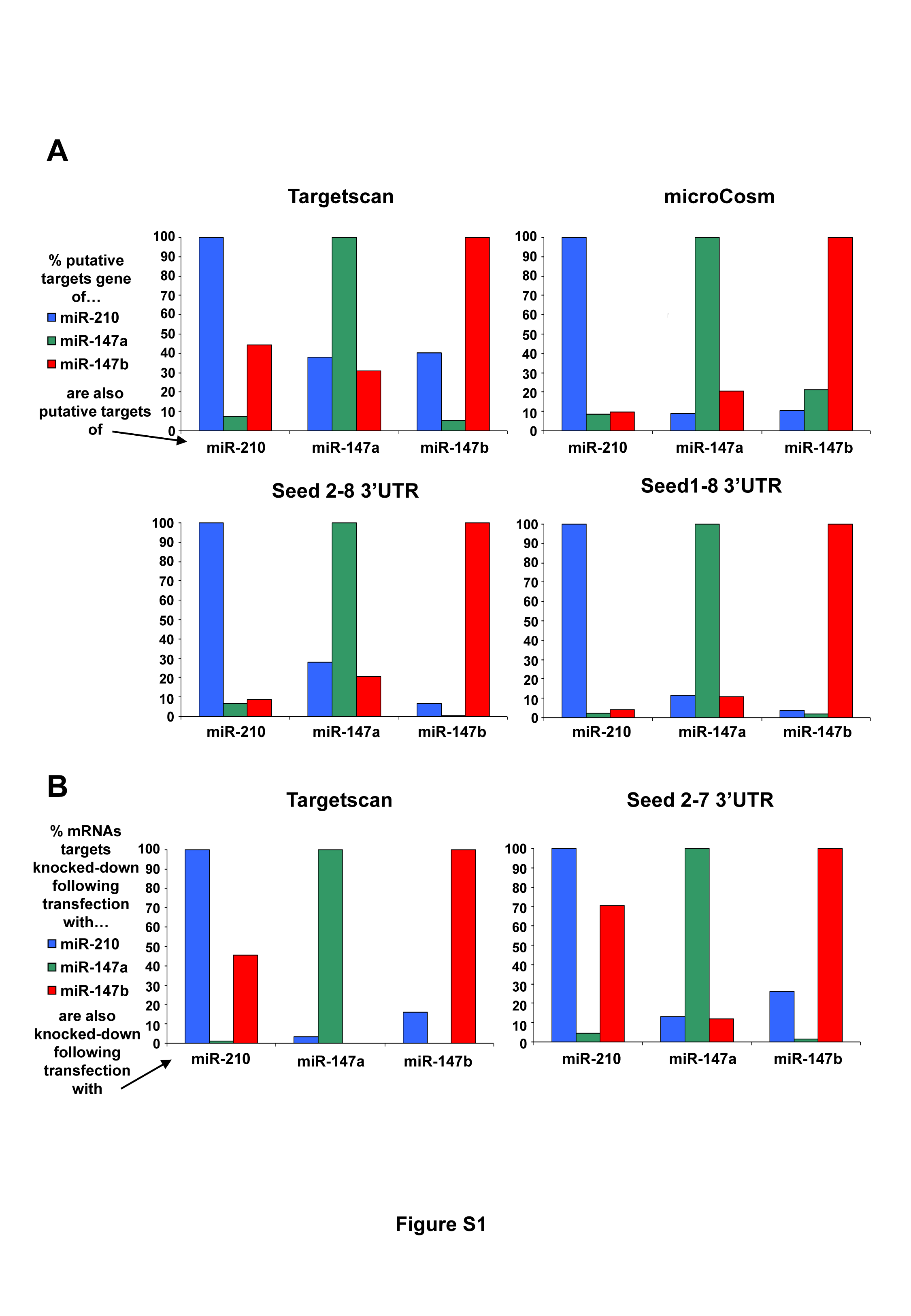

Supplement: Figure S1 — Overlap between predicted targets of miR-210 and miR-147 family members. A) In silico evaluation of the common predicted targets between hsa-miR-210, hsa-miR-147a and hsa-miR-147b using TargetScan or microCosm. B) Overlap between the predicted targets for each of the 3 miRNAs that are significantly down-regulated following transfection by each of the miRNA candidates. Data were calculated using our webtool miRontop (Lebrigand et al. 2010, Bioinformatics) using the following cut offs : log2Average>8; logFC<-0,5 and Adj.pVal<0,05. Note that an important percentage of genes knocked down by miR-210 were also knocked down by miR-147b but not by miR-147a. (TIF) [file pone.0044919.s001.tif]

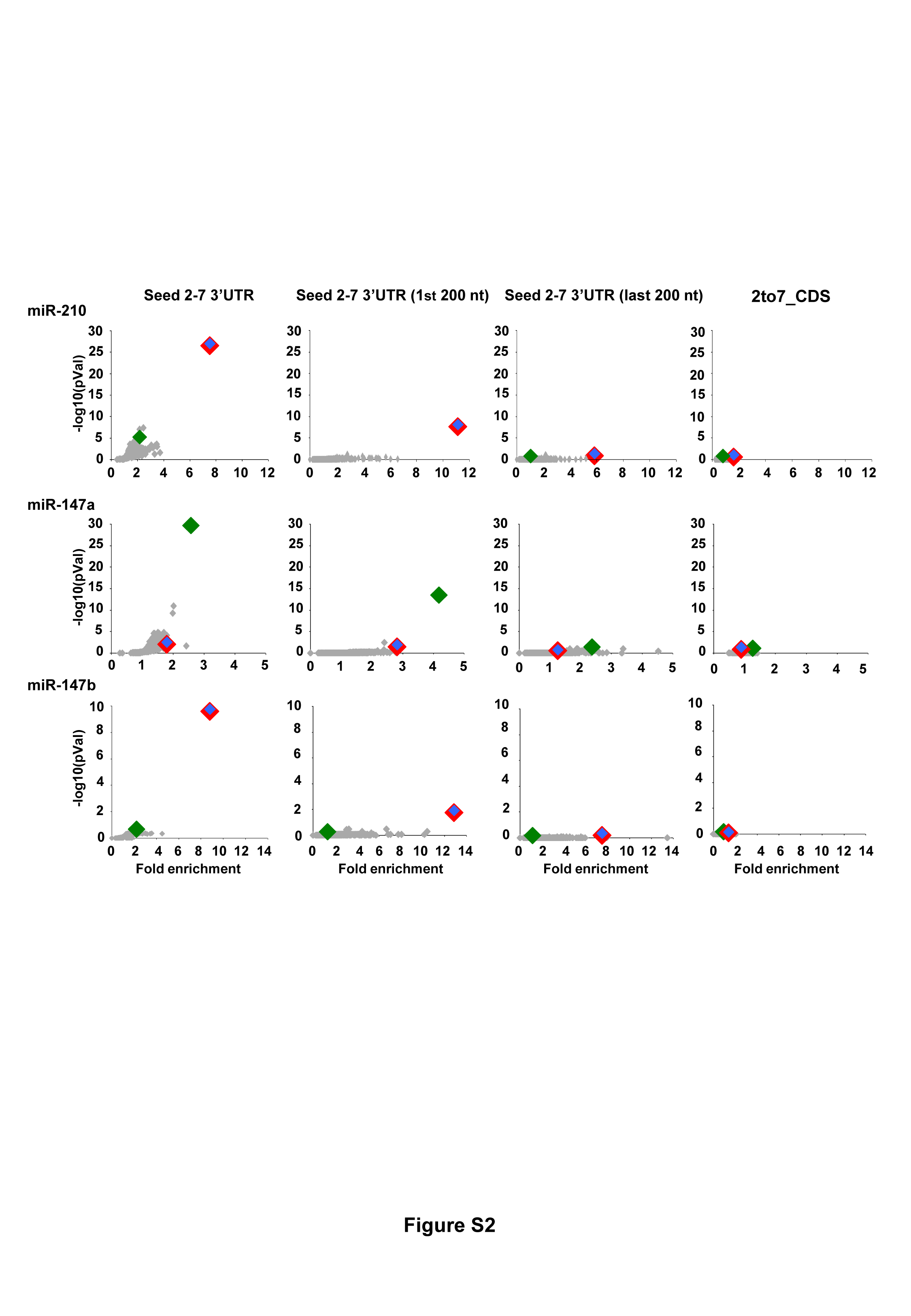

Supplement: Figure S2 — Graphs adapted from our webtool miRontop (Le Brigand et al. 2010, Bioinformatics) showing the significance of the enrichment (represented as –log10 (adjPVal) according to the fold enrichment in experiments of overexpression of hsa-miR-210, hsa-miR-147a and hsa-miR-147b. Enrichment was calculated according to a 2–7 seed search in distinct regions of the transcripts. On each panel, hsa-miR-210, hsa-miR-147a and hsa-miR-147b are highlighted as blue, green and red dots, respectively. (TIF) [file pone.0044919.s002.tif]

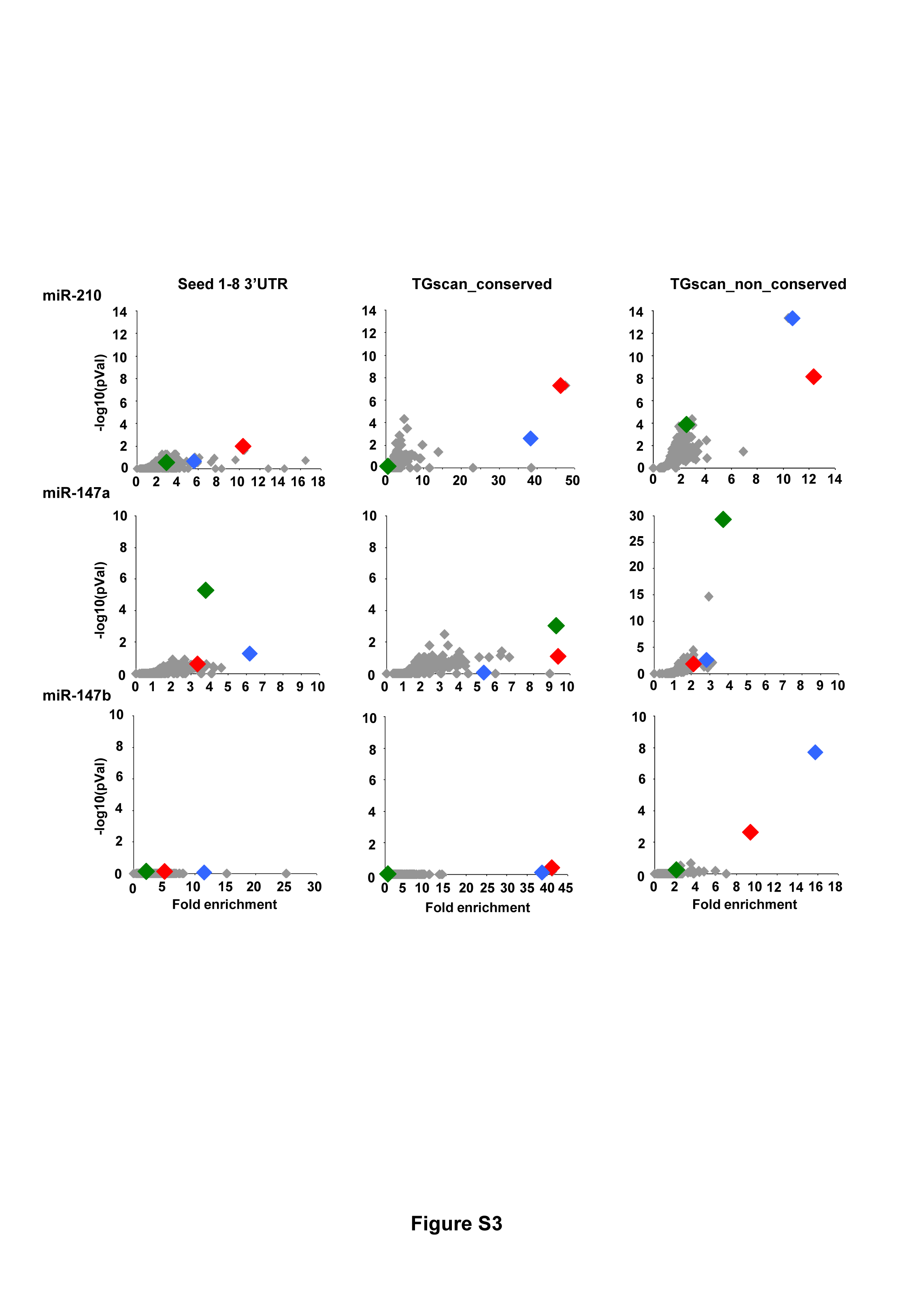

Supplement: Figure S3 — Graphs adapted from our webtool miRontop (Le Brigand et al. 2010, Bioinformatics) showing the significance of the enrichment (represented as –log10 (adjPVal) according to the fold enrichment in experiments of overexpression of hsa-miR-210, hsa-miR-147a and hsa-miR-147b. Enrichment was calculated according to a 1–8 seed search in 3′UTR or using the conserved or non-conserved miRNA targets prediction database from TargetScan. On each panel, hsa-miR-210, hsa-miR-147a and hsa-miR-147b are highlighted as blue, green and red dots, respectively. (TIF) [file pone.0044919.s003.tif]

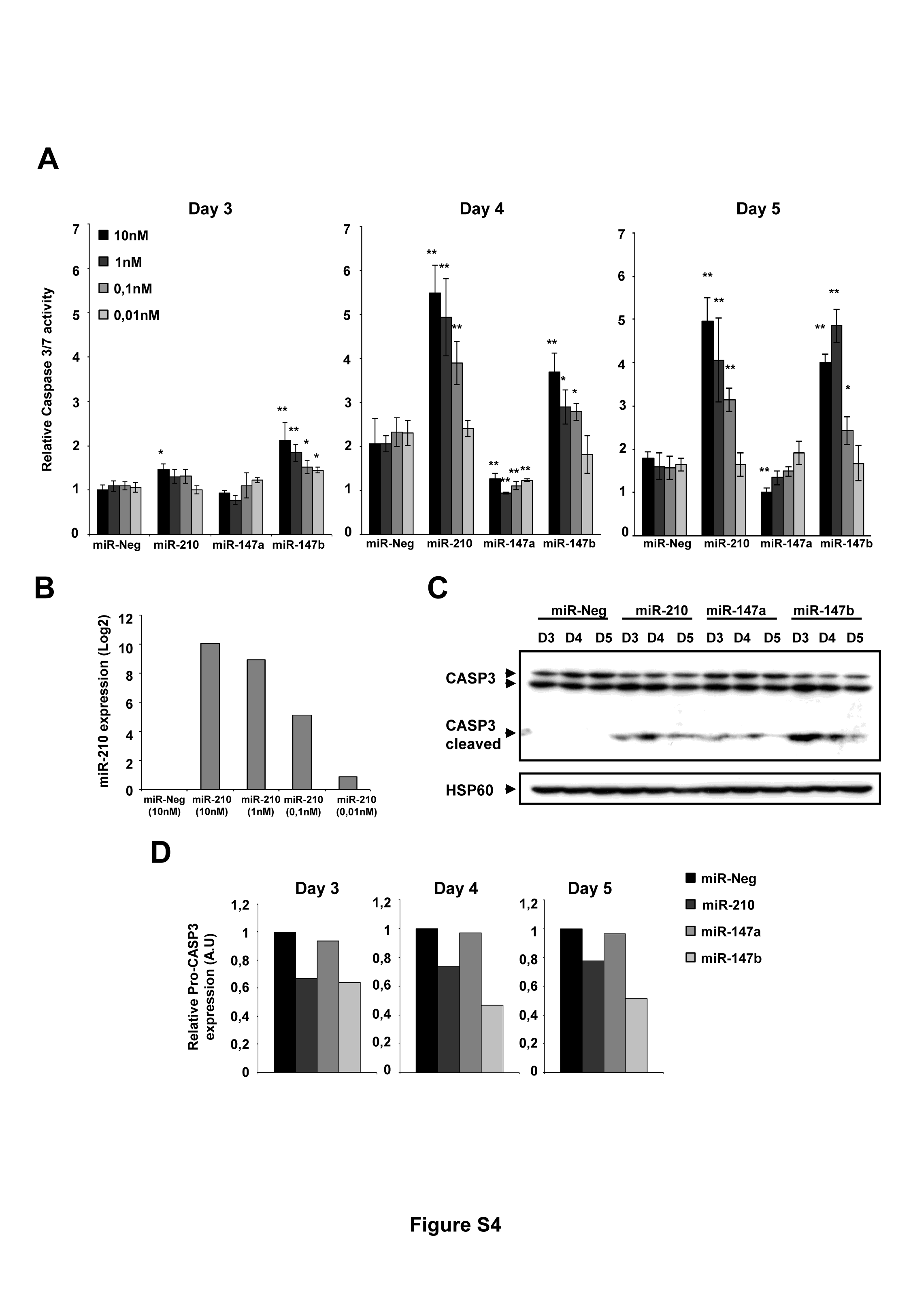

Supplement: Figure S4 — Dose-response effect of miR-210, miR-147a and miR-147b on A459 cells viability. A549 cells were transfected with 10 nM, 1nM, 0,1nM or 0,01nM of hsa-pre-miR-210, hsa-pre-miR-147a, hsa-pre-miR-147b or pre-miR-Neg and analyzed for several viability parameters. A) Caspase 3/7 assay was performed at 3, 4 and 5 days after transfection. Data are mean ± SD values of 2 independent experiments performed in triplicate. B) Cells were collected 48 h after transfection and the relative miR-210 levels were determined using a TaqMan assay. C) Expression of pro-caspase-3 was analyzed by Western blot in A549 cells transfected with each indicated pre-miRNA at 10 nM. Hsp60 corresponds to the loading control. D) Densitometric quantification of pro-caspase-3 gene normalized for Hsp60 signal. (TIF) [file pone.0044919.s004.tif]

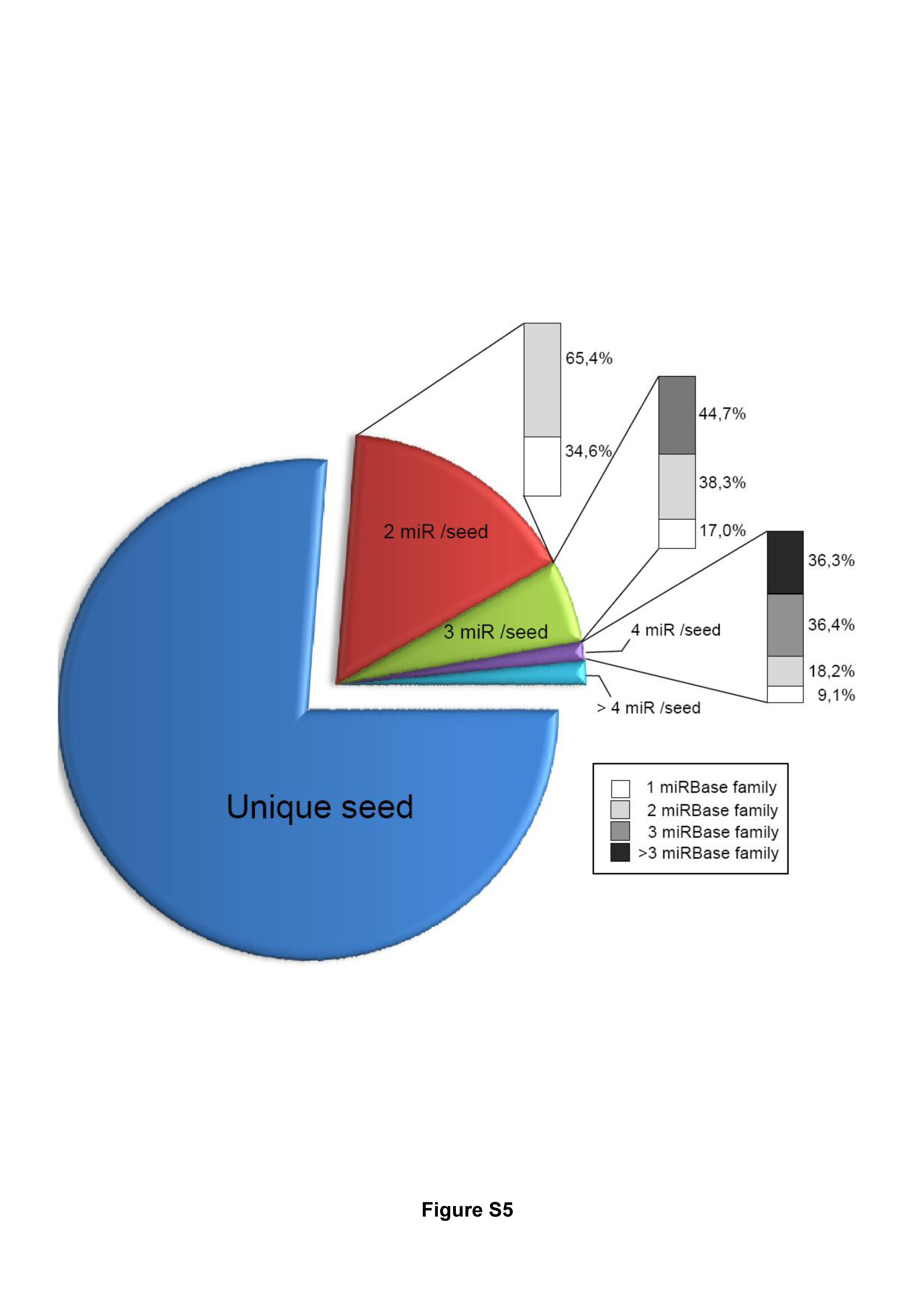

Supplement: Figure S5 — Usage of distinct seed (2–7 nt) sequences among human miRNAs. Pie-chart showing the representation of seed 2–7 among all human mature miRNAs. On the 872 distinct human seed sequences in miRBase v16, 665 are unique and 207 are shared by two or more miRNAs (miR). MiRNAs sharing the same seed sequence can belong to distinct miRBase families, thus the number of distinct miRBase families was reported for each shared seed sequence. The proportion of represented miRBase family in the shared seeds is shown as a barplot for the seeds shared by 2, 3 and 4 miRNAs. (TIF) [file pone.0044919.s005.tif]

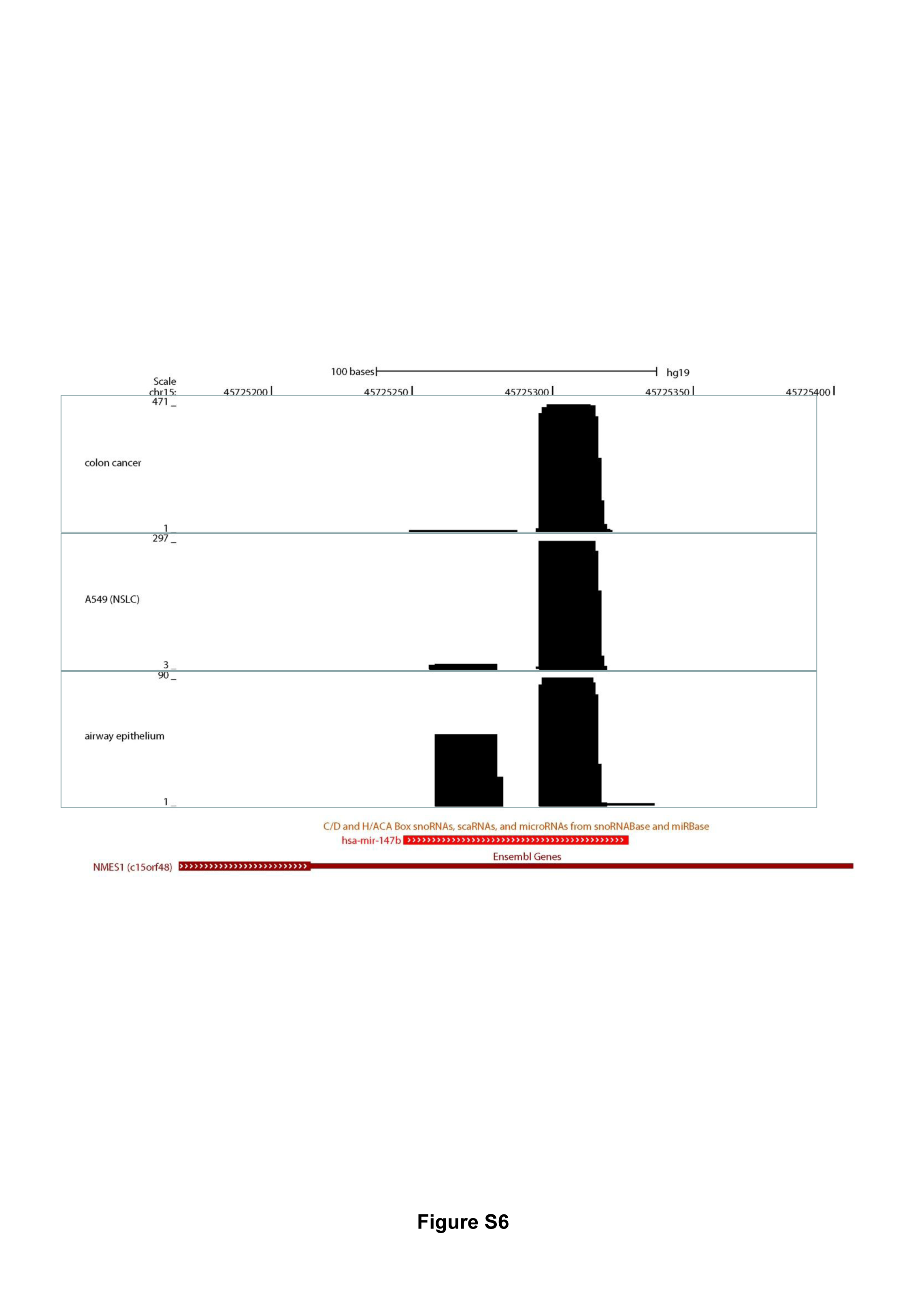

Supplement: Figure S6 — Expression of hsa-miR-147b in different human tissues using Small RNA Seq. Screenshot of the human miR-147b locus generated by the UCSC genome browser (hg19 assembly). Black boxes correspond to coverage of each base position (bigwig files). Data from 3 human samples were loaded, with sample description on the left part of the tracks. Annotated transcripts of the locus, including miR-147b, are shown at the bottom (red box with arrows showing the strand direction). Total RNA were isolated from colon cancer, Non Small Cell Lung Cancer cell line A549 and normal airway epithelial cells (obtained from inferior turbinates from patients who underwent surgical intervention for nasal obstruction). The SOLiD™ Small RNA Expression Kit (Applied Biosystems, Life Technologies Corporation) was used to build a library of double-stranded DNA molecules from the population of small RNAs present in the different samples, which were then read using the Applied Biosystems SOLiD™ System sequencing according to the manufacturer’s instructions. Libraries were amplified by emulsion PCR and sequenced on SOLiD according to the manufacturer’s instructions. Read length was 35 bp. Color-space reads were matched against annotated databases using the Small RNA Analysis Pipeline Tool v5.0 (RNA2MAP), provided by Applied Biosystems, using the following parameters: one color-space mismatch within the first 18 bases of the reads, called the ‘seed sequence’ and two color-space mismatches on the following positions of the reads. Reads were matched against the human genome (hg19). (TIF) [file pone.0044919.s006.tif]
