# Supplementary material for: “Seed-Milarity” Confers to hsa-miR-210 and hsa-miR-147b Similar Functional Activity
Source: PLoS One. 2012 Sep 13;7(9):e44919. doi: 10.1371/journal.pone.0044919 (PMC3441733; doi:10.1371/journal.pone.0044919)
Supplement: Table S1 — List of forward and reverse primers used for cloning 3′UTR sequences in pSI-CHECKTM-2 (Promega) by cloning behind the Renilla luciferase in the XhoI and NotI restrictions sites. (DOCX) [file pone.0044919.s007.docx]

| **Gene** | **Forward** | **Reverse** |
| --- | --- | --- |
| ALDH5A1 | CGACCTGAGCCTGAGTAAGTGG | AACCCAGAATAACCACAAGATGTAAGTG |
| BDKRB2 | GCAAACGCCAGCAGGGCTG | GCACCAAAGCTGGATGGCAGTTCT |
| COL4A2 | GGAATTTGCATCCAGCAGCAGCAC | CCTCTGGCCACGGCTGGC |
| E2F3 | ACTACTCGAGGTGTTGTCCCTTCCTACCTTCTT | ATAAGAATGCGGCCGCGCACATTTTCTCACTTGCTGAC |
| EHD2 | CCTGCTGTGGCTCCCCAG | GGGGCTGGGGGCCCTG |
| FOXN3 | GGTGCCAGGAAAGCCACTGGA | GGCAGGTTCCGTAGCTTTCTAG |
| IER5 | GGCTCATTGGAAGAGGACGATCG | TGCGGGGAAGCAAAGGACCG |
| IGFBP3 | GCACAGCACCCAGACTTCATGC | GAGGGCCCGAAATACCTGCC |
| INPP5A | CTCTGCCGATGCTCCTTCCG | GTTGTAACGTAGGATACTTGGCACC |
| MAPK6 | CTTCCCCTCAAATTCCTCATCAAAC | GCATTTCTTTTCTCCTCCTGCG |
| MCM3 | CAGGACTCGGTGGACCCTTTGG | CTTGACCTGCATGACGTGCCTC |
| PSMA4 | CAAGAGAGAATGGAAAGACAGTAATCAG | CTGCTACAGAGAATTTATTCCTTCATTTCC |
| NDUFA4 | GAAATGTTTCACTATAACGCTGCTTTAG | GCCAGTGTCAGATGCTGGAG |
| SDHD | GGTGGACAGCCTTCTTCTCTTAATC | CATAGAATACATTTTCACATTAGAGATTCCC |
| SH3BGRL | CCTGTCTCTCATTCCTAAGTTGCCT | GCTTACTTGGCATCCCACTTCATG |
