# Supplementary material for: “Seed-Milarity” Confers to hsa-miR-210 and hsa-miR-147b Similar Functional Activity
Source: PLoS One. 2012 Sep 13;7(9):e44919. doi: 10.1371/journal.pone.0044919 (PMC3441733; doi:10.1371/journal.pone.0044919)
Supplement: Table S2 — List of themes corresponding to “Molecular Function” annotations identified by Ingenuity Pathway Analysis in response to overexpression of hsa-miR-210, hsa-mir-147a or hsa-miR-147b. The probability to obtain the number of genes in a certain pathway in the list of differentially expressed genes was compared with the representation of the same pathway among all the genes on the microarray and was calculated as a Fisher’s exact probability (p-value cut-off = 0.001). (DOCX) [file pone.0044919.s008.docx]

| **Category** | **-LOG(p-value)** | | |
| --- | --- | --- | --- |
|  | **Obs1 (miR-147a)** | **Obs2 (miR-147b)** | **Obs3 (miR-210)** |
| Amino Acid Metabolism | 2.91 | 2.26 | 3.41 |
| Antigen Presentation | NS | 3.09 | 2.16 |
| Carbohydrate Metabolism | 3.79 | 2.69 | 2.95 |
| Cell Cycle | 15.09 | 3.36 | 6.61 |
| Cell Death | 5.59 | 3.42 | 5.91 |
| Cell Morphology | 4.21 | 2.80 | 2.52 |
| Cell Signaling | NS | 2.34 | 3.64 |
| Cell-To-Cell Signaling and Interaction | 2.44 | 4.26 | 3.23 |
| Cellular Assembly and Organization | 11.55 | 2.32 | 3.41 |
| Cellular Compromise | 2.78 | 2.26 | 2.11 |
| Cellular Development | 3.39 | 5.19 | 5.22 |
| Cellular Function and Maintenance | 4.37 | 2.32 | 3.41 |
| Cellular Growth and Proliferation | 5.74 | 4.26 | 5.12 |
| Cellular Movement | 3.94 | 4.05 | 5.24 |
| DNA Replication, Recombination, and Repair | 11.55 | 2.26 | 2.95 |
| Drug Metabolism | 3.78 | 2.26 | 2.42 |
| Energy Production | 2.84 | NS | NS |
| Free Radical Scavenging | NS | 2.12 | NS |
| Lipid Metabolism | 4.14 | 2.26 | 3.16 |
| Molecular Transport | 2.91 | 2.34 | 3.41 |
| Nucleic Acid Metabolism | 3.10 | 2.26 | 2.64 |
| Post-Translational Modification | 2.72 | 2.69 | 3.41 |
| Protein Synthesis | 2.72 | NS | 1.89 |
| Protein Trafficking | NS | NS | 2.30 |
| RNA Post-Transcriptional Modification | 2.24 | NS | 2.16 |
| Small Molecule Biochemistry | 4.14 | 2.69 | 3.41 |
| Vitamin and Mineral Metabolism | 4.14 | 2.34 | 2.55 |
