# Supplementary material for: “Seed-Milarity” Confers to hsa-miR-210 and hsa-miR-147b Similar Functional Activity
Source: PLoS One. 2012 Sep 13;7(9):e44919. doi: 10.1371/journal.pone.0044919 (PMC3441733; doi:10.1371/journal.pone.0044919)
Supplement: Table S3 — List of themes corresponding to “canonical pathways” annotations identified by Ingenuity Pathway Analysis in response to overexpression of hsa-miR-210, hsa-miR-147a or hsa-miR-147b. The probability to obtain the number of genes in a certain pathway in the list of differentially expressed genes was compared with the representation of the same pathway among all the genes on the microarray and was calculated as a Fisher’s exact probability (p-value cut-off = 0.05). The genes modulated in each theme are represented. (DOCX) [file pone.0044919.s009.docx]

| **Ingenuity Canonical Pathways** | **-LOG(p-value)** | | |
| --- | --- | --- | --- |
|  | **Obs1 (miR-147a)** | **Obs2 (miR-147b)** | **Obs3 (miR-210)** |
| Acute Phase Response Signaling | 1.16 | 0.64 | 0.94 |
| Aldosterone Signaling in Epithelial Cells | 0.35 | 0.69 | 2.46 |
| Aminosugars Metabolism | 1.44 | ns | 1.30 |
| Apoptosis Signaling | 0.42 | 0.41 | 1.50 |
| Ascorbate and Aldarate Metabolism | 0.32 | ns | 2.30 |
| ATM Signaling | 3.38 | ns | ns |
| Biosynthesis of Steroids | 2.25 | ns | ns |
| Butanoate Metabolism | 0.84 | ns | 2.24 |
| Butanoate Metabolism | ns | ns | ns |
| Calcium-induced T Lymphocyte Apoptosis | 0.43 | 0.54 | 2.86 |
| CDK5 Signaling | 0.44 | 1.09 | 1.03 |
| Cell Cycle Control of Chromosomal Replication | 3.54 | ns | ns |
| Cell Cycle: G1/S Checkpoint Regulation | 1.34 | ns | 0.52 |
| Cell Cycle: G2/M DNA Damage Checkpoint Regulation | 3.11 | ns | 0.23 |
| Chondroitin Sulfate Biosynthesis | 1.55 | 0.63 | 0.60 |
| Chronic Myeloid Leukemia Signaling | 1.33 | ns | 0.54 |
| Clathrin-mediated Endocytosis Signaling | 0.96 | ns | ns |
| Cleavage and Polyadenylation of Pre-mRNA | ns | ns | 2.83 |
| CNTF Signaling | 1.46 | ns | ns |
| Cyclins and Cell Cycle Regulation | 2.76 | ns | 1.14 |
| Death Receptor Signaling | 0.82 | 1.38 | 2.97 |
| DNA Double-Strand Break Repair by Homologous Recombination | 1.07 | ns | ns |
| DNA Methylation and Transcriptional Repression Signaling | 0.81 | 0.98 | 2.17 |
| EGF Signaling | 1.66 | ns | 0.64 |
| EIF2 Signaling | 2.04 | ns | 0.63 |
| Endoplasmic Reticulum Stress Pathway | 0.89 | 1.03 | 1.31 |
| Endothelin-1 Signaling | 0.28 | 0.64 | 1.33 |
| Ephrin Receptor Signaling | 1.74 | ns | 0.59 |
| Glucocorticoid Receptor Signaling | 0.88 | 0.76 | 2.21 |
| HMGB1 Signaling | ns | 1.83 | 1.41 |
| IGF-1 Signaling | 0.87 | 0.38 | 1.33 |
| IL-17 Signaling | ns | 1.23 | 2.52 |
| IL-8 Signaling | ns | 1.16 | 2.12 |
| ILK Signaling | ns | 0.57 | 1.12 |
| Inositol Phosphate Metabolism | 0.70 | ns | 1.27 |
| Integrin Signaling | 1.33 | 0.53 | 0.70 |
| JAK/Stat Signaling | 1.70 | ns | 0.92 |
| Lysine Degradation | 1.20 | ns | 1.49 |
| Mismatch Repair in Eukaryotes | 4.97 | ns | ns |
| Mitochondrial Dysfunction | 4.14 | 2.21 | 0.89 |
| Mitotic Roles of Polo-Like Kinase | 2.90 |  | 2.15 |
| mTOR Signaling | 0.63 | 0.74 | 1.62 |
| Nicotinate and Nicotinamide Metabolism | 0.87 | 0.38 | 1.33 |
| NRF2-mediated Oxidative Stress Response | 0.98 | 0.59 | 1.16 |
| O-Glycan Biosynthesis | 1.13 | ns | ns |
| Oncostatin M Signaling | 1.52 | ns | ns |
| Oxidative Phosphorylation | 4.96 | 1.36 | 0.50 |
| p38 MAPK Signaling | 1.48 | 0.36 |  |
| p38 MAPK Signaling | ns | ns | ns |
| p53 Signaling | 2.27 | ns | 1.43 |
| Pentose Phosphate Pathway | 1.00 | ns | ns |
| Phospholipase C Signaling | ns | ns | 1.39 |
| Phospholipid Degradation | ns | 1.20 | 0.36 |
| Production of Nitric Oxide and Reactive Oxygen Species in Macrophages | 0.34 | 0.69 | 1.47 |
| Protein Ubiquitination Pathway | 0.51 | 0.38 | 2.22 |
| Purine Metabolism | 1.90 | ns | ns |
| Pyrimidine Metabolism | 2.94 | 0.76 | 0.29 |
| Rac Signaling | 2.45 | ns | 0.82 |
| RAN Signaling | 2.71 | ns | 0.56 |
| RAN Signaling | ns | ns | ns |
| RAR Activation | 0.27 | 0.22 | 1.74 |
| Regulation of Actin-based Motility by Rho | 1.13 | 0.43 | 0.63 |
| Regulation of eIF4 and p70S6K Signaling | 1.12 | ns | ns |
| Role of CHK Proteins in Cell Cycle Checkpoint Control | 3.05 | ns | ns |
| Role of IL-17F in Allergic Inflammatory Airway Diseases | 0.35 | 2.78 | 1.27 |
| Role of JAK1, JAK2 and TYK2 in Interferon Signaling | 1.30 | ns | ns |
| Role of JAK2 in Hormone-like Cytokine Signaling | 1.48 | ns | ns |
| Role of Tissue Factor in Cancer | 0.47 | 0.92 | 1.67 |
| Sphingosine-1-phosphate Signaling | ns | 0.93 | 1.20 |
| TGF-β Signaling | 0.25 | 0.44 | 1.61 |
| Thrombopoietin Signaling | 1.37 | ns | ns |
| Tight Junction Signaling | ns | 0.24 | 1.90 |
| TNFR1 Signaling | 0.30 | 0.64 | 1.16 |
| TREM1 Signaling | 0.92 | 1.45 | 1.04 |
| Ubiquinone Biosynthesis | 2.02 | 2.21 | 0.41 |
| VDR/RXR Activation | 0.29 | ns | 1.16 |
