# Supplementary material for: “Seed-Milarity” Confers to hsa-miR-210 and hsa-miR-147b Similar Functional Activity
Source: PLoS One. 2012 Sep 13;7(9):e44919. doi: 10.1371/journal.pone.0044919 (PMC3441733; doi:10.1371/journal.pone.0044919)
Supplement: Table S4 — Full list of the predicted targets transcripts down-regulated following hsa-miR-210, hsa-miR-147a or hsa-miR-147b overexpression in A549 cells. The transcripts predicted to be targets (seed 2–7 in 3′UTR) are highlited. Logarithm (base 2) of the average expression, logarithm (base 2) of the ratio of miR-210/miR-Neg and false discovery rate p-values using the Benjamini-Hochberg correction are represented. ID: correspond to RNG oligo IDs that give access to transcripts and probes annotations through our information system Mediante (http://www.microarray.fr:8080/merge/index). (DOCX) [file pone.0044919.s010.docx]

| **ID** | **Name** | **AveExpr** | **logFC 210** | **logFC 147a** | **logFC 147b** | **adj.P.Val 210** | **adj.P.Val 147a** | **adj.P.Val 147b** |
| --- | --- | --- | --- | --- | --- | --- | --- | --- |
| 39961 | ADIPOR2 | 9.16 | -0.45 | -0.92 | -0.57 | 2.39E-01 | 1.91E-03 | 1.63E-01 |
| 13964 | AHCYL1 | 10.90 | -0.16 | -1.10 | -0.16 | 9.01E-01 | 1.37E-04 | 9.20E-01 |
| 79713 | AHNAK2 | 11.25 | -0.43 | -0.57 | -0.40 | 7.14E-02 | 7.95E-03 | 1.96E-01 |
| 63840 | ALDH5A1 | 8.52 | -0.67 | 0.08 | -0.45 | 1.28E-03 | 9.32E-01 | 5.42E-02 |
| 105748 | ANTXR1 | 10.29 | -0.39 | -0.87 | -0.21 | 6.98E-03 | 1.07E-06 | 3.32E-01 |
| 4607 | ARHGAP1 | 10.71 | 0.08 | -0.51 | 0.05 | 9.12E-01 | 4.78E-04 | 9.88E-01 |
| 9719 | ARPC2 | 12.30 | -0.01 | -0.82 | 0.01 | 1.00E+00 | 6.75E-05 | 9.97E-01 |
| 70301 | ATP11A | 8.22 | -0.79 | -0.51 | -0.34 | 6.27E-03 | 6.63E-02 | 5.66E-01 |
| 90132 | ATP1A1 | 10.33 | -0.02 | -0.53 | -0.01 | 1.00E+00 | 1.17E-04 | 9.97E-01 |
| 8423 | B4GALT5 | 10.92 | -0.61 | -0.53 | -0.30 | 4.47E-03 | 6.90E-03 | 3.84E-01 |
| 6139 | BAG2 | 9.33 | -0.41 | -0.75 | -0.23 | 2.09E-01 | 3.84E-03 | 7.85E-01 |
| 90051 | BDKRB2 | 8.89 | -0.51 | -0.31 | -0.35 | 1.42E-02 | 1.60E-01 | 2.33E-01 |
| 89252 | BID | 8.51 | 0.00 | -0.63 | 0.05 | 1.00E+00 | 1.02E-02 | 9.95E-01 |
| 41566 | C16orf53 | 9.99 | 0.14 | -0.69 | 0.00 | 8.41E-01 | 1.22E-03 | 9.99E-01 |
| 41452 | C1orf116 | 8.32 | -0.59 | -0.23 | -0.25 | 7.47E-03 | 4.49E-01 | 5.86E-01 |
| 50575 | C6orf153 | 10.24 | 0.13 | -0.65 | 0.04 | 8.73E-01 | 1.91E-03 | 9.94E-01 |
| 14363 | CALM1 | 11.22 | -0.06 | -1.28 | 0.06 | 1.00E+00 | 2.07E-03 | 9.97E-01 |
| 92991 | CAPN2 | 12.39 | -0.07 | -0.52 | -0.11 | 9.88E-01 | 6.59E-03 | 9.38E-01 |
| 14227 | CBX1 | 12.30 | 0.07 | -1.50 | 0.11 | 9.84E-01 | 2.96E-07 | 9.43E-01 |
| 65561 | CCDC34 | 9.82 | 0.25 | -0.51 | 0.14 | 3.51E-01 | 6.54E-03 | 8.68E-01 |
| 47812 | CCDC97 | 9.32 | -0.49 | -0.70 | -0.17 | 5.91E-02 | 2.71E-03 | 8.64E-01 |
| 13180 | CD164 | 10.66 | -0.43 | -0.68 | -0.31 | 1.13E-02 | 8.62E-05 | 1.59E-01 |
| 18516 | CD24 | 13.65 | -0.02 | -0.78 | 0.05 | 1.00E+00 | 2.74E-04 | 9.90E-01 |
| 79008 | CDK6 | 9.85 | 0.31 | -1.13 | 0.11 | 2.70E-01 | 7.89E-06 | 9.49E-01 |
| 28859 | CEP55 | 9.45 | -0.10 | -0.98 | 0.00 | 9.38E-01 | 1.57E-05 | 9.99E-01 |
| 22758 | CEP57 | 8.95 | 0.11 | -0.55 | 0.01 | 8.68E-01 | 2.07E-03 | 9.97E-01 |
| 85082 | CHMP4B | 10.57 | -0.56 | 0.07 | -0.54 | 1.07E-03 | 9.28E-01 | 2.87E-03 |
| 112388 | CHP | 11.20 | -0.42 | -1.03 | -0.43 | 4.56E-02 | 7.89E-06 | 8.38E-02 |
| 6134 | CHST3 | 9.86 | -0.17 | -0.58 | -0.01 | 7.06E-01 | 3.35E-03 | 9.97E-01 |
| 94674 | COL4A2 | 10.51 | -1.51 | 0.01 | -0.96 | 2.39E-04 | 9.99E-01 | 2.22E-02 |
| 20832 | CORO1C | 11.64 | -0.52 | -0.28 | -0.85 | 1.09E-01 | 5.51E-01 | 1.17E-02 |
| 40216 | COTL1 | 11.20 | -0.33 | -1.01 | -0.20 | 3.66E-01 | 1.63E-04 | 8.30E-01 |
| 12925 | CTDSPL | 9.28 | 0.14 | -0.91 | 0.09 | 7.78E-01 | 8.23E-06 | 9.52E-01 |
| 72084 | DCBLD1 | 9.76 | -1.14 | -0.26 | -1.13 | 4.00E-04 | 6.13E-01 | 1.21E-03 |
| 9043 | DDOST | 10.31 | -0.73 | 0.26 | -0.25 | 9.39E-03 | 5.73E-01 | 7.65E-01 |
| 19427 | DIMT1L | 9.43 | -0.83 | 0.01 | -0.38 | 5.20E-05 | 9.98E-01 | 5.91E-02 |
| 25295 | dJ222E13.2 | 10.68 | 0.10 | -0.52 | 0.05 | 9.36E-01 | 5.10E-03 | 9.90E-01 |
| 17277 | DNASE1L1 | 9.53 | -0.23 | -0.85 | -0.06 | 1.78E-01 | 2.62E-06 | 9.76E-01 |
| 28177 | DYM | 9.07 | -0.18 | -0.78 | -0.12 | 7.96E-01 | 1.50E-03 | 9.52E-01 |
| 45021 | ECHDC3 | 8.55 | -0.17 | -0.74 | -0.24 | 8.35E-01 | 2.65E-03 | 7.55E-01 |
| 22648 | EHD2 | 10.06 | -0.87 | -0.86 | -0.41 | 6.15E-03 | 3.12E-03 | 4.82E-01 |
| 146787 | EIF2S3 | 12.53 | -0.17 | -0.67 | -0.17 | 8.02E-01 | 3.35E-03 | 8.70E-01 |
| 114977 | EIF5 | 12.35 | -0.06 | -0.77 | 0.05 | 9.98E-01 | 5.04E-04 | 9.91E-01 |
| 957 | ELFN2 | 8.29 | -0.64 | -0.24 | -0.41 | 1.37E-02 | 5.61E-01 | 2.80E-01 |
| 83501 | EMD | 10.76 | 0.04 | -0.53 | 0.05 | 1.00E+00 | 1.83E-03 | 9.89E-01 |
| 174127 | EPB41L1 | 10.03 | -0.76 | 0.15 | -0.46 | 4.52E-04 | 7.06E-01 | 5.13E-02 |
| 27537 | EVL | 10.00 | 0.10 | -0.73 | 0.07 | 9.36E-01 | 2.99E-04 | 9.86E-01 |
| 69086 | FAM102A | 9.41 | -0.52 | -0.22 | -0.65 | 2.19E-02 | 5.58E-01 | 1.17E-02 |
| 116003 | FAM3C | 8.87 | -0.88 | -0.34 | -0.71 | 1.28E-03 | 2.72E-01 | 1.58E-02 |
| 28209 | FERMT1 | 9.22 | -0.10 | -0.64 | 0.03 | 9.65E-01 | 4.25E-03 | 9.97E-01 |
| 32402 | FLJ11151 | 9.53 | 0.02 | -0.66 | -0.10 | 1.00E+00 | 1.27E-03 | 9.49E-01 |
| 49505 | FLJ42258 | 10.73 | 0.36 | -1.11 | 0.28 | 3.20E-01 | 7.80E-05 | 6.86E-01 |
| 37840 | FNDC3B | 9.63 | 0.13 | -0.57 | -0.02 | 6.39E-01 | 1.37E-04 | 9.95E-01 |
| 94153 | FOXF2 | 8.34 | -0.03 | -0.58 | -0.02 | 1.00E+00 | 4.62E-04 | 9.97E-01 |
| 4872 | FOXK2 | 9.06 | -0.10 | -0.83 | -0.06 | 9.35E-01 | 6.69E-05 | 9.88E-01 |
| 12135 | FOXN3 | 8.36 | -0.54 | 0.08 | -0.36 | 2.45E-03 | 8.99E-01 | 7.93E-02 |
| 20367 | FTSJ2 | 10.61 | -0.24 | -0.53 | -0.21 | 5.42E-01 | 1.77E-02 | 7.70E-01 |
| 36665 | GALNT1 | 10.26 | -0.06 | -0.52 | -0.10 | 9.89E-01 | 2.54E-03 | 9.37E-01 |
| 104909 | GIT2 | 9.01 | -0.74 | -0.05 | -0.57 | 6.98E-03 | 9.87E-01 | 7.58E-02 |
| 11279 | GPR56 | 11.02 | -0.74 | 0.23 | -0.63 | 6.09E-04 | 3.91E-01 | 6.23E-03 |
| 11719 | HDAC4 | 8.19 | -0.54 | 0.07 | -0.36 | 5.43E-03 | 9.43E-01 | 1.30E-01 |
| 8048 | HDGF | 10.12 | -0.02 | -0.57 | 0.00 | 1.00E+00 | 1.22E-03 | 9.99E-01 |
| 156049 | HIPK2 | 9.82 | -0.56 | -0.78 | -0.82 | 1.80E-01 | 2.36E-02 | 5.50E-02 |
| 99398 | HLTF | 9.69 | 0.09 | -0.62 | 0.02 | 9.34E-01 | 4.78E-04 | 9.97E-01 |
| 11012 | HMGN2 | 13.28 | -0.01 | -1.14 | 0.00 | 1.00E+00 | 1.03E-05 | 9.99E-01 |
| 138030 | HNRPDL | 11.09 | 0.10 | -0.62 | 0.18 | 9.46E-01 | 3.05E-03 | 8.09E-01 |
| 11017 | HOXA1 | 8.17 | -0.60 | -0.07 | -0.36 | 3.10E-03 | 9.42E-01 | 1.68E-01 |
| 11027 | HSF1 | 10.00 | 0.13 | -0.66 | -0.06 | 9.45E-01 | 1.13E-02 | 9.91E-01 |
| 172706 | HSPA8 | 14.56 | 0.16 | -0.75 | 0.10 | 4.74E-01 | 7.43E-06 | 8.75E-01 |
| 171749 | IARS | 12.36 | 0.03 | -0.66 | -0.06 | 1.00E+00 | 4.11E-03 | 9.90E-01 |
| 137631 | IER5 | 11.55 | -0.33 | -0.37 | -0.64 | 5.37E-02 | 1.72E-02 | 1.13E-03 |
| 88461 | IGFBP3 | 9.56 | -1.32 | -0.34 | -0.95 | 4.08E-03 | 7.09E-01 | 7.50E-02 |
| 92770 | IL13RA1 | 9.97 | 0.04 | -0.83 | -0.30 | 1.00E+00 | 8.62E-04 | 5.82E-01 |
| 12584 | INPP5A | 9.14 | -0.75 | -0.60 | -0.28 | 2.65E-03 | 7.83E-03 | 5.82E-01 |
| 5640 | IQGAP1 | 10.16 | 0.15 | -0.72 | 0.12 | 5.41E-01 | 7.89E-06 | 7.78E-01 |
| 19216 | ISCU | 10.96 | -1.12 | 0.08 | -0.63 | 4.05E-05 | 9.51E-01 | 1.44E-02 |
| 113953 | ISOC1 | 8.87 | 0.23 | -0.69 | 0.14 | 5.80E-01 | 2.05E-03 | 9.06E-01 |
| 17040 | KHDRBS1 | 12.18 | 0.08 | -0.63 | 0.15 | 8.30E-01 | 8.04E-06 | 6.01E-01 |
| 22964 | KIAA0329 | 8.33 | -0.53 | -0.50 | -0.54 | 1.43E-02 | 1.29E-02 | 2.51E-02 |
| 24332 | KIAA0406 | 8.80 | 0.07 | -0.54 | 0.03 | 9.95E-01 | 1.30E-02 | 9.97E-01 |
| 25424 | KIAA0859 | 9.14 | -0.64 | -0.03 | -0.41 | 3.36E-04 | 9.86E-01 | 2.52E-02 |
| 170771 | KIF23 | 9.24 | -0.03 | -0.66 | 0.04 | 1.00E+00 | 3.08E-05 | 9.89E-01 |
| 21502 | KPNA6 | 9.67 | -0.03 | -0.69 | -0.06 | 1.00E+00 | 3.88E-03 | 9.90E-01 |
| 95248 | KPNB1 | 13.94 | -0.21 | -1.35 | 0.01 | 8.23E-01 | 3.86E-05 | 9.97E-01 |
| 14926 | LASP1 | 13.04 | -1.11 | -0.52 | -0.60 | 1.12E-03 | 1.25E-01 | 1.54E-01 |
| 26571 | LEPROTL1 | 9.49 | -0.57 | -0.04 | -0.31 | 2.12E-03 | 9.77E-01 | 2.01E-01 |
| 9776 | LHFP | 8.40 | -0.95 | -1.07 | -0.70 | 4.33E-02 | 1.29E-02 | 3.01E-01 |
| 82990 | LHFPL2 | 10.66 | 0.07 | -0.85 | -0.05 | 9.33E-01 | 1.20E-06 | 9.81E-01 |
| 66007 | LOC205251 | 10.22 | 0.02 | -1.00 | 0.03 | 1.00E+00 | 6.85E-05 | 9.97E-01 |
| 26227 | LPGAT1 | 9.50 | 0.08 | -0.54 | 0.03 | 8.88E-01 | 1.42E-04 | 9.91E-01 |
| 1729 | LRRC8A | 10.88 | -0.69 | 0.03 | -0.86 | 6.29E-03 | 9.95E-01 | 2.17E-03 |
| 28755 | MAP7D1 | 10.61 | -0.01 | -0.57 | 0.06 | 1.00E+00 | 3.08E-03 | 9.89E-01 |
| 98960 | MAPK6 | 10.05 | 0.35 | -0.65 | 0.24 | 1.02E-01 | 8.62E-04 | 5.73E-01 |
| 93821 | MCM3 | 9.18 | 0.02 | -0.92 | -0.13 | 1.00E+00 | 2.50E-04 | 9.42E-01 |
| 128026 | MCM8 | 8.33 | -0.37 | -0.57 | -0.18 | 1.81E-01 | 1.19E-02 | 8.25E-01 |
| 19545 | MGLL | 9.64 | -0.40 | -0.54 | -0.22 | 7.99E-02 | 7.70E-03 | 6.94E-01 |
| 48403 | MRPL36 | 10.97 | -0.60 | -0.11 | -0.26 | 2.02E-02 | 9.16E-01 | 7.01E-01 |
| 26419 | MRPS27 | 11.51 | -0.71 | -1.79 | -0.38 | 4.57E-02 | 7.77E-06 | 6.43E-01 |
| 27766 | MST4 | 8.81 | -0.33 | -0.71 | -0.36 | 2.41E-01 | 1.54E-03 | 3.02E-01 |
| 65858 | MUTED | 8.74 | 0.20 | -0.57 | 0.11 | 2.83E-01 | 1.53E-04 | 8.43E-01 |
| 93928 | MYD88 | 10.75 | -0.05 | -1.27 | 0.07 | 1.00E+00 | 7.77E-06 | 9.89E-01 |
| 18351 | MYO10 | 11.00 | 0.03 | -0.71 | -0.04 | 1.00E+00 | 3.07E-05 | 9.89E-01 |
| 50953 | #NA | 9.42 | -0.71 | -0.54 | -0.77 | 9.81E-03 | 3.85E-02 | 1.34E-02 |
| 10879 | NCOA4 | 11.86 | 0.02 | -0.60 | -0.10 | 1.00E+00 | 7.20E-04 | 9.41E-01 |
| 93962 | NDUFA4 | 12.78 | -1.75 | -2.17 | -2.15 | 7.14E-03 | 5.04E-04 | 2.87E-03 |
| 38340 | NKAP | 9.60 | 0.05 | -0.72 | 0.05 | 1.00E+00 | 3.36E-04 | 9.90E-01 |
| 86418 | NP | 11.47 | 0.04 | -1.66 | 0.07 | 1.00E+00 | 6.85E-05 | 9.95E-01 |
| 14959 | NSF | 10.27 | 0.05 | -0.87 | 0.16 | 9.97E-01 | 7.77E-06 | 7.24E-01 |
| 42162 | NUP210 | 10.08 | -0.71 | -0.22 | -0.42 | 3.23E-03 | 5.82E-01 | 1.98E-01 |
| 26101 | NUPL1 | 8.99 | 0.43 | -0.72 | 0.38 | 9.60E-03 | 3.61E-05 | 4.37E-02 |
| 164949 | OPRS1 | 9.82 | -0.22 | -1.30 | -0.41 | 8.91E-01 | 5.04E-04 | 6.45E-01 |
| 35282 | OSBPL8 | 9.59 | 0.18 | -0.57 | 0.09 | 4.18E-01 | 1.63E-04 | 9.04E-01 |
| 40015 | OSBPL9 | 9.87 | -0.05 | -0.76 | -0.21 | 1.00E+00 | 2.50E-04 | 6.87E-01 |
| 13738 | PAICS | 11.71 | 0.02 | -1.15 | -0.19 | 1.00E+00 | 2.64E-05 | 8.30E-01 |
| 37342 | PDXK | 10.60 | -0.57 | -0.38 | -0.44 | 2.37E-03 | 2.93E-02 | 3.57E-02 |
| 74015 | PHF19 | 9.40 | -0.22 | -0.81 | -0.13 | 7.40E-01 | 2.36E-03 | 9.61E-01 |
| 12409 | PODXL | 10.50 | 0.47 | -0.59 | 0.57 | 8.13E-03 | 6.24E-04 | 3.98E-03 |
| 95803 | PPP1CC | 12.49 | 0.04 | -0.52 | 0.12 | 1.00E+00 | 2.25E-03 | 8.79E-01 |
| 13476 | PPP1R2 | 8.30 | -0.86 | 0.07 | -0.34 | 9.11E-03 | 9.79E-01 | 6.64E-01 |
| 163543 | PRICKLE4 | 11.42 | -0.47 | -0.51 | -1.01 | 7.31E-02 | 3.50E-02 | 1.10E-03 |
| 95914 | PSMA4 | 11.99 | -0.25 | -1.05 | 0.02 | 7.97E-01 | 1.84E-03 | 9.97E-01 |
| 50403 | PTPN11 | 10.75 | 0.03 | -0.95 | 0.06 | 1.00E+00 | 3.43E-05 | 9.89E-01 |
| 97563 | PTPRK | 10.52 | 0.02 | -0.70 | 0.01 | 1.00E+00 | 7.89E-06 | 9.97E-01 |
| 96046 | RAD23B | 10.95 | 0.03 | -1.47 | -0.05 | 1.00E+00 | 1.31E-04 | 9.97E-01 |
| 34545 | RBM47 | 10.00 | -0.33 | -0.51 | -0.52 | 2.44E-01 | 1.86E-02 | 5.42E-02 |
| 94397 | RHOG | 9.23 | 0.06 | -0.61 | 0.08 | 9.69E-01 | 6.85E-05 | 9.43E-01 |
| 152539 | RHOT1 | 9.04 | -0.31 | -0.16 | -0.68 | 3.39E-01 | 8.00E-01 | 1.44E-02 |
| 38858 | RIC8A | 10.48 | -0.06 | -0.94 | -0.28 | 1.00E+00 | 6.37E-04 | 6.97E-01 |
| 34168 | RNF130 | 10.02 | -0.07 | -1.48 | 0.04 | 9.97E-01 | 1.20E-06 | 9.95E-01 |
| 30769 | RNF138 | 9.29 | 0.22 | -0.67 | 0.12 | 4.31E-01 | 4.29E-04 | 9.07E-01 |
| 96106 | RNF4 | 9.88 | 0.16 | -0.50 | -0.02 | 7.41E-01 | 7.89E-03 | 9.97E-01 |
| 170520 | RTN4 | 12.85 | -0.02 | -0.66 | 0.00 | 1.00E+00 | 1.03E-05 | 9.99E-01 |
| 24194 | SCARA3 | 9.31 | -1.11 | -1.04 | -0.79 | 1.39E-02 | 1.39E-02 | 2.00E-01 |
| 17519 | SDF2 | 8.75 | -0.83 | 0.15 | -0.82 | 5.71E-03 | 8.82E-01 | 1.44E-02 |
| 18235 | SEPHS2 | 11.28 | -0.18 | -0.59 | -0.10 | 3.22E-01 | 3.86E-05 | 8.55E-01 |
| 72098 | SFXN2 | 8.36 | -0.11 | -0.63 | -0.05 | 9.17E-01 | 1.72E-03 | 9.90E-01 |
| 97796 | SH3BGRL | 9.75 | -0.91 | 0.27 | -0.34 | 1.87E-04 | 2.97E-01 | 2.71E-01 |
| 99331 | SH3GL1 | 10.72 | -0.65 | -0.89 | -0.25 | 5.11E-02 | 3.41E-03 | 8.25E-01 |
| 91599 | SLC31A1 | 9.09 | 0.16 | -1.32 | 0.16 | 8.86E-01 | 8.04E-06 | 9.07E-01 |
| 109585 | SLC44A1 | 10.10 | -0.11 | -0.93 | -0.42 | 9.40E-01 | 6.40E-05 | 1.42E-01 |
| 21740 | SLCO3A1 | 8.59 | 0.03 | -0.51 | 0.02 | 1.00E+00 | 1.24E-03 | 9.97E-01 |
| 99431 | SNX2 | 9.69 | 0.01 | -0.69 | 0.09 | 1.00E+00 | 4.97E-05 | 9.27E-01 |
| 97923 | SPARC | 12.11 | 0.05 | -0.58 | 0.13 | 9.98E-01 | 7.80E-04 | 8.42E-01 |
| 18893 | SPCS1 | 11.70 | -0.06 | -0.76 | -0.10 | 9.98E-01 | 1.89E-04 | 9.49E-01 |
| 156931 | SPRED2 | 8.73 | -0.59 | 0.01 | -0.24 | 5.40E-04 | 9.98E-01 | 3.16E-01 |
| 69 | SRGAP2 | 9.25 | -0.11 | -0.61 | -0.05 | 8.40E-01 | 4.04E-04 | 9.88E-01 |
| 97955 | SRPK1 | 10.14 | -0.16 | -0.61 | -0.15 | 7.94E-01 | 4.08E-03 | 8.80E-01 |
| 37093 | SRPRB | 10.25 | 0.14 | -0.80 | 0.10 | 7.70E-01 | 5.56E-05 | 9.44E-01 |
| 96396 | SSR1 | 9.85 | 0.02 | -1.48 | -0.03 | 1.00E+00 | 6.60E-06 | 9.97E-01 |
| 16234 | SUPT16H | 9.87 | -0.57 | -0.02 | -0.71 | 5.43E-03 | 9.98E-01 | 2.17E-03 |
| 36797 | SYT13 | 9.46 | 0.38 | -0.53 | 0.14 | 6.50E-02 | 3.86E-03 | 8.59E-01 |
| 98011 | TAPBP | 9.76 | -0.03 | -0.76 | -0.04 | 1.00E+00 | 4.05E-04 | 9.95E-01 |
| 36068 | TERF2IP | 10.04 | -0.81 | -0.19 | -0.40 | 9.13E-04 | 6.56E-01 | 2.10E-01 |
| 78533 | TMEM185B | 8.61 | 0.04 | -0.54 | 0.02 | 1.00E+00 | 2.25E-03 | 9.97E-01 |
| 17190 | TPBG | 9.55 | 0.17 | -0.51 | 0.20 | 6.31E-01 | 2.91E-03 | 6.32E-01 |
| 46704 | TRAF7 | 10.48 | -0.33 | -0.80 | -0.09 | 4.73E-01 | 4.38E-03 | 9.88E-01 |
| 6094 | TRIP13 | 10.03 | 0.19 | -0.91 | 0.12 | 7.34E-01 | 1.73E-04 | 9.45E-01 |
| 13151 | TSPAN31 | 9.81 | -0.61 | 0.12 | -0.45 | 1.12E-03 | 7.75E-01 | 2.49E-02 |
| 13557 | TYRO3 | 8.75 | -0.13 | -0.52 | -0.18 | 9.08E-01 | 2.01E-02 | 8.26E-01 |
| 58813 | UBLCP1 | 9.54 | 0.25 | -0.95 | 0.38 | 7.42E-01 | 1.60E-03 | 5.60E-01 |
| 5205 | VAPB | 9.83 | 0.05 | -0.84 | 0.05 | 1.00E+00 | 8.92E-04 | 9.94E-01 |
| 25764 | VPS28 | 9.32 | 0.04 | -0.67 | 0.02 | 1.00E+00 | 7.74E-04 | 9.97E-01 |
| 2756 | WWC1 | 9.58 | -0.62 | -0.58 | -0.52 | 3.02E-03 | 2.36E-03 | 2.49E-02 |
| 13560 | XRCC1 | 9.04 | 0.07 | -0.57 | 0.05 | 9.34E-01 | 1.63E-04 | 9.88E-01 |
| 37575 | XYLT2 | 9.36 | 0.32 | -0.64 | 0.33 | 3.21E-02 | 3.61E-05 | 5.68E-02 |
| 61149 | ZNF664 | 10.75 | -0.05 | -1.49 | -0.14 | 1.00E+00 | 1.03E-05 | 9.60E-01 |
